# 3D microelectrode cluster and stimulation paradigm yield powerful analgesia without noticeable adverse effects

**DOI:** 10.1126/sciadv.abj2847

**Published:** 2021-10-08

**Authors:** Matilde Forni, Palmi Thor Thorbergsson, Jonas Thelin, Jens Schouenborg

**Affiliations:** 1 Neuronano Research Center, Department of Experimental Medical Sciences, Medical Faculty, Lund University, Medicon Village, Scheelevägen 2, Lund, 223 81, Sweden.; 2 NanoLund, Center for Nanoscience, Lund University, Professorsgatan 1, Lund 223 63, Sweden.

## Abstract

The lack of satisfactory treatment for persistent pain profoundly impairs the quality of life for many patients. Stimulation of brainstem pain control systems can trigger powerful analgesia, but their complex network organization frequently prevents separation of analgesia from side effects. To overcome this long-standing challenge, we developed a biocompatible gelatin-embedded cluster of ultrathin microelectrodes that enables fine-tuned, high-definition three-dimensional stimulation in periaqueductal gray/dorsal raphe nucleus in awake rats. Analgesia was assessed from both motor reactions and intracortical signals, corresponding to pain-related signals in humans. We could select an individual-specific subset of microelectrodes in each animal that reliably provided strong pain inhibition during normal and hyperalgesia conditions, without noticeable behavioral side effects. Gait, spontaneous cortical activity at rest, and cortical tactile responses were minimally affected, indicating a highly selective action. In conclusion, our developed biocompatible microelectrode cluster and stimulation paradigm reliably enabled powerful, fine-tuned, and selective analgesia without noticeable side effects.

## Teaser

Powerful pain relief without noticeable adverse effects is attainable with high-definition granular brain stimulation.

## INTRODUCTION

Persistent pain continues to be a major scientific and clinical challenge, despite considerable efforts to develop effective analgesic treatments. Yet, powerful pain control systems exist in the brain. These systems involve the periaqueductal gray substance (PAG) and dorsal raphe nucleus (DRN), which exerts strong control over nociceptive spinal systems (*1*–*4*) via descending connections. PAG and DRN are complex nuclei in the mesencephalon involved in orchestrating several complex behavioral responses in life-threatening situations (*5*–*7*) and also have subregions that can elicit strong inhibition of pain reactions (*8*–*11*) upon electrical stimulation in awake animals and provide analgesia in patients (*12*–*18*). Stimulation of different neuron types in the PAG/DRN induces different circuit effects. For example, dopamine neurons in this region have been shown to produce analgesia without anxiety (*19*). In addition, different microcircuits of γ-aminobutyric acid (GABA) and glutamate neurons with divergent end targets can produce competing behaviors (*20*). One function of this powerful control may be to prevent pain-related reactions from interfering with other, more urgent, and behaviorally appropriate actions.

Several attempts to use this powerful analgesic control to treat patients with intractable pain have been made by implanting electrodes for deep brain stimulation (DBS) within PAG/DRN. However, the success rate is variable and unreliable (*21*–*24*). Analgesic effects are often mixed with clinically notable adverse effects, ranging from anxiety or panic, vertigo or nausea, and gaze problems (*13*, *14*, *25*–*28*). Plausible reasons for this outcome include the following: (i) the large size of the electrodes relative to target structures, producing widespread currents that also may stimulate nearby neuronal networks involved in, for example, autonomic or emotional responses; (ii) improper placement of the electrodes; or (iii) positional instability (migration) of the implanted electrodes in the tissue. A contributing factor may be that the electrodes used so far, while stiff enough to be precisely implanted, are known to cause a loss of nearby neurons and elicit substantial glial, “foreign body” reactions (*29*–*32*) that can result in a fibrous encapsulation of the implanted electrode (*33*–*35*). These tissue reactions, in turn, make higher stimulation currents necessary to reach viable nervous tissue and thus increase the risk of current spread to unwanted regions (*36*). Most likely, these tissue responses contribute to the common finding of deteriorating therapeutic effects of stimulation over time (*21*).

Given that highly flexible and thin microelectrodes cause less severe tissue reactions than rigid ones (*37*–*41*) and offer a possibility for highly precise stimulation, they may help overcome the frequently occurring adverse effects of state-of-the-art simulation-based treatments of persistent pain. However, because thin microelectrodes have a limited capacity to eject current, the accuracy of their placements is critical. This situation is problematic because the brain coordinates of optimal stimulation sites are not fully known and may even differ between patients because of small individual anatomical variations. Moreover, the precise placement of ultraflexible microelectrodes into deep targets presents another challenge. Hence, it is not clear whether microelectrode stimulation has the potential of providing a reliable and powerful analgesic therapy.

**Fig. 1.**
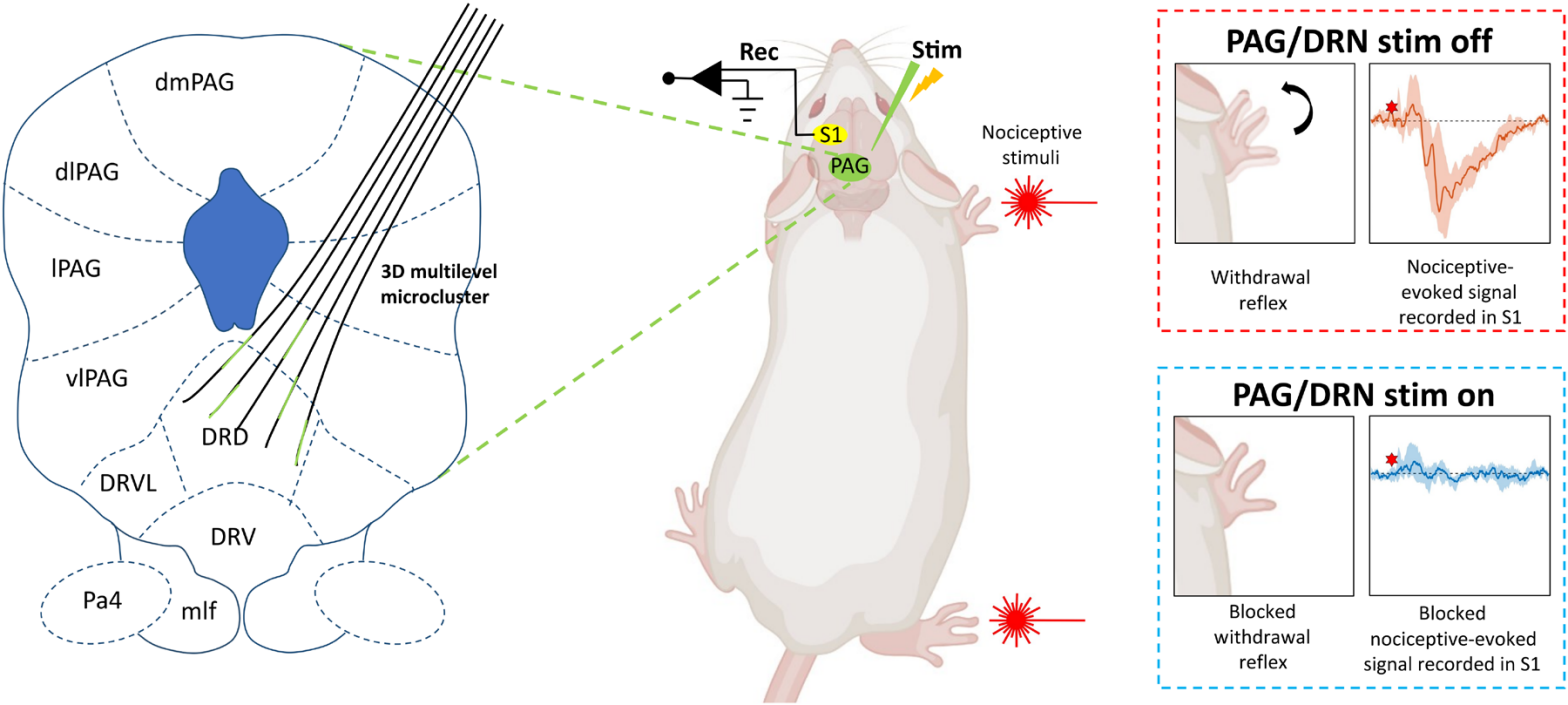
Overview of methods. **Left:** Schematic of a subset of implanted microelectrodes in PAG/DRN in a coronal plane. The green lines represent the electrode contacts for stimulation distributed in 3D. **Middle:** Awake and freely moving rat chronically implanted with stimulation probe in PAG/DRN and recording array in S1 cortex. Nociceptive stimuli were delivered to the paws contralateral to the implant in the S1 cortex. **Right:** Intracortical recordings [only evoked field potential (FP) shown] and reflex assessments during nociceptive stimulation in control and PAG/DRN stimulation conditions. dmPAG, dorsomedial PAG; dlPAG, dorsolateral PAG; lPAG, lateral PAG; vlPAG, ventrolateral PAG; DRD, the dorsal subregion of the DRN; DRV, the ventral subregion of the DRN; DRVL, ventrolateral subregion of the DRN; Pa4, paratrochlear nucleus; mlf, medial longitudinal fasciculus (the rat illustration was created with BioRender.com).

**Fig. 2.**
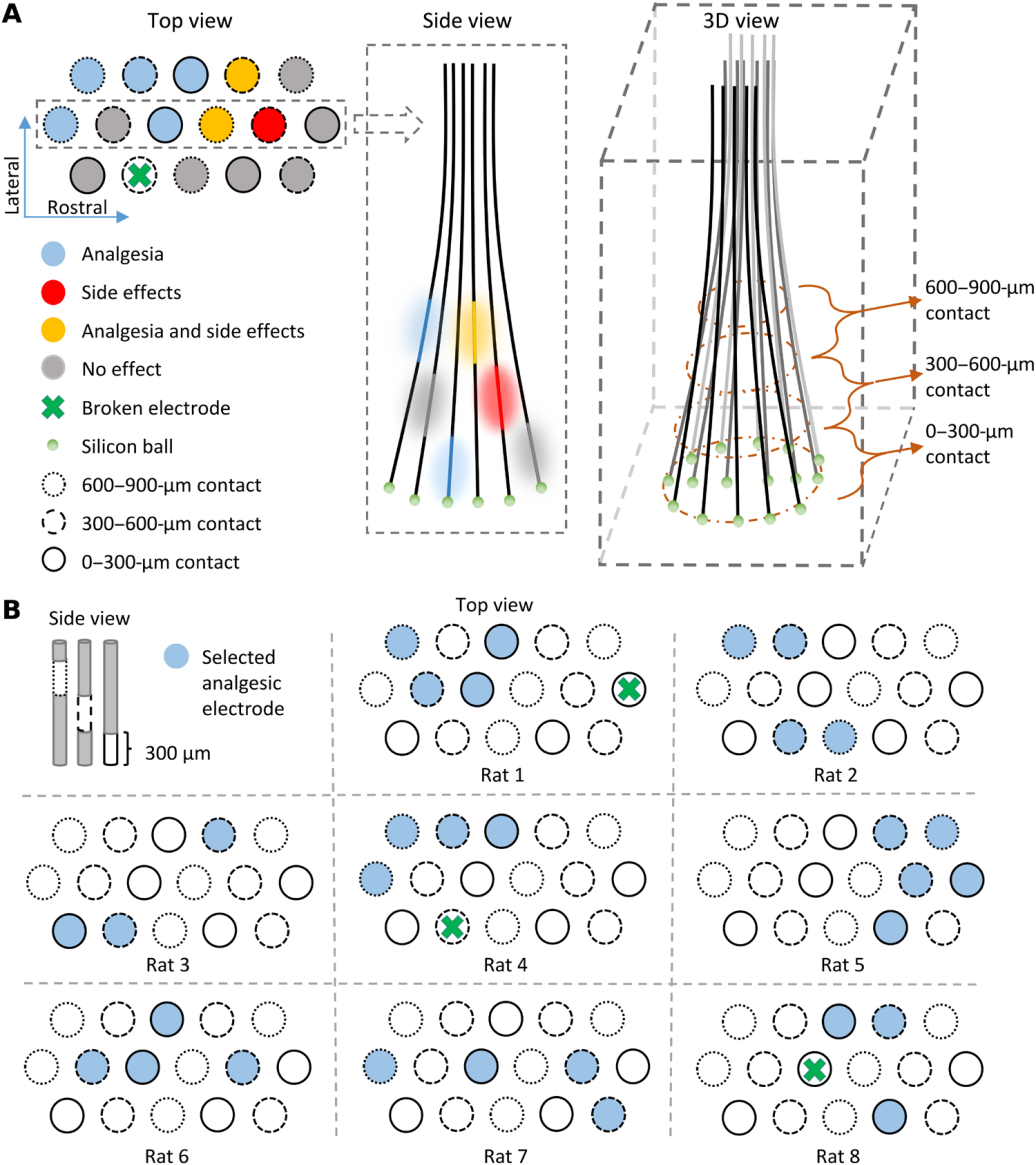
Schematic of microelectrode cluster and electrode selection. (**A**) Left: Top view of a microelectrode cluster. Each circle represents a single microelectrode, and a green cross signifies a nonfunctional microelectrode. The location/level of stimulation sites along the microelectrodes and stimulation effect of each microelectrode is indicated by contours and colors, respectively. Middle: Side view of the stimulation probe. Stimulation sites and fields are shown schematically by colored lines and patches, respectively. Distalmost green circles represent silicon blobs used to eliminate sharp damaging contours at the tip of the electrode. Right: 3D view of the stimulation probe. Circles indicate the boundaries between three different levels of stimulation sites. (**B**) The panels represent the top view of selected microelectrodes (in blue) that together induced reflex analgesia in all four paws in the respective animal (*n* = 8).

To address the problem of accomplishing precise brain stimulation with minimal tissue reactions, we have previously developed methods to insert highly flexible and ultrathin microelectrodes into deep brain targets (*42*). By embedding the microelectrodes in needle-shaped, hard gelatin that expands and then dissolves during implantation, highly flexible microelectrodes could be implanted and spread out as a cluster in deep brain targets (*43*, *44*). This design was further developed in the present study by introducing a multilevel location of the deinsulated contacts (Figs. 1 and 2), with the intention to provide stimulation sites in a volume including both PAG and DRN and thereby enable a novel paradigm for fine-tuned, granulated stimulation of neural tissue in three dimensions (3D). Notably, a cluster design also reduces the problems of not knowing in advance the precise location of appropriate stimulation sites.

The main aim of the present study was to clarify whether reliable stimulation-induced analgesia, without noticeable adverse effects, can be accomplished by selecting in each rat appropriate microelectrodes from a cluster of highly flexible microelectrodes implanted in both caudal PAG and DRN. To evaluate the analgesic effects in a translational animal model of pain, we made use of previous findings indicating that nociceptive-evoked activity recorded in the primary somatosensory cortex (S1) in awake freely moving rats mimics key aspects of pain perception in humans during control conditions and hyperalgesia (*45*–*47*). To this end, highly flexible microelectrodes were also implanted in the hindlimb and forelimb S1 cortex for recordings of nociceptive-evoked field potentials (FPs) and neuronal responses. In addition, conventional assessments of nociceptive withdrawal reflexes and normal motor performance (gait) were made to gauge the presence of analgesia and side effects (Fig. 1). We report that strong and highly specific analgesia, with no sign of sedation or adverse behavioral effects, can indeed be accomplished by stimulating highly specific subregions of neural tissue through an appropriate individual subset of microelectrodes selected from a flexible microelectrode cluster implanted in PAG/DRN.

## RESULTS

### Individualized microelectrode and stimulation parameter selection

To identify individually appropriate subsets of microelectrodes, yielding antinociceptive effects without noticeable side effects, we performed a selection procedure analyzing implanted microelectrode clusters in eight animals with verified PAG/DRN implantation [computed tomography (CT) or histological analysis showing a resulting placement within or close to PAG/DRN according to the standard Waxholm rat atlas of PAG used; fig. S1] during weeks 2 to 3 postsurgery (PS). Initially, the effects of single microelectrode stimulation on reflex responses to nociceptive heat stimulation (CO_2_ laser pulses) of the paws were evaluated using currents of maximum 50 μA and a frequency of 50 Hz (Fig. 2A). At least 2 of the 16 implanted microelectrodes (median 5) were found to inhibit nociceptive reflexes elicited from at least one paw in each animal. Side effects were elicited by 1 to 11 microelectrodes (median 3), mixed effects were elicited by 0 to 7 microelectrodes (median 1.5), and the remainder caused no clear effects. On the basis of this initial screening, combinations of potentially useful microelectrodes were tested in each animal until stimulation through a combination of three to five microelectrodes (median 4) abolished nociceptive reflexes in all four paws, without noticeable behavioral effects. In this search process, combinations of microelectrodes that produced side effects were not further used, thus narrowing down the number of viable options. Typical side effects included stereotypic head movements, rotations, backward steps, jumps, urination, searching behaviors, heavy breathing, and behavioral signs of alertness (i.e., increased vigilance). For each animal, the successful combination of microelectrodes included stimulation sites at different distances from the distalmost tips (Fig. 2B). The stimulation current for this selection of microelectrodes was then further adjusted such that reflex responses were abolished without noticeable adverse effects. This current strength was defined in each animal and termed as *I*_max_ (median current per microelectrode was 40 μA). For each animal, the individually selected subset of microelectrodes and current strengths were thereafter kept unchanged for the rest of the study, except when evaluating the effects of lower currents than Imax.

**Fig. 3.**
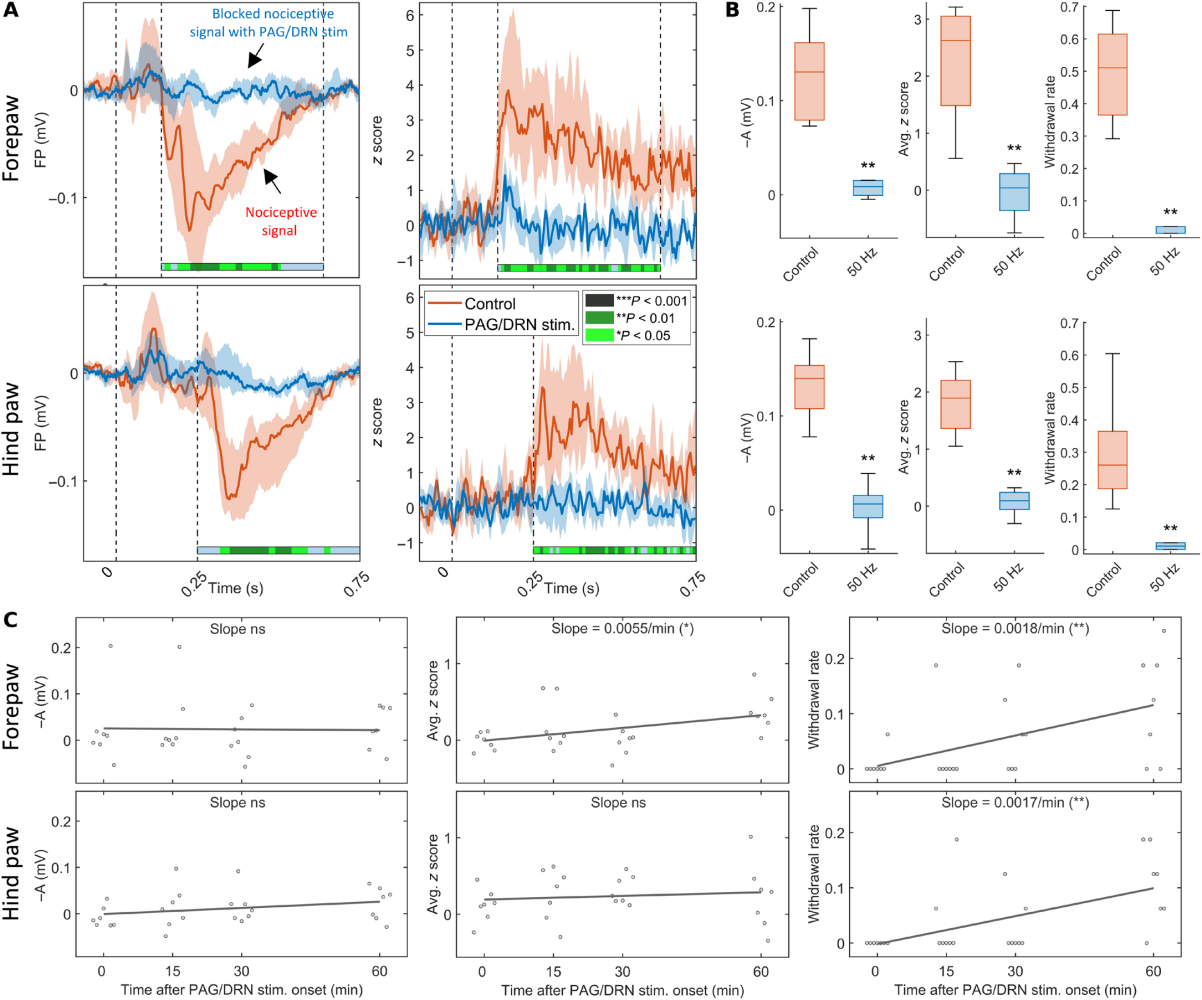
Abolished nociceptive-evoked FP and neuronal responses in primary somatosensory cortex and nociceptive withdrawal reflexes during PAG/DRN stimulation. (**A**) The colored lines indicate the median (*n* = 8) of nociceptive-evoked FP amplitude (left) and neuronal responses *z* score (right), in control and during PAG/DRN stimulation (Imax, 50 Hz) recorded in contralateral fore- and hind paw S1. The shaded area around the median values represents the interquartile range. The recordings are shown from −0.1 to 0.750 s with respect to stimulus onset. The horizontal color-coded bars under each graph represent the statistical significance level (Wilcoxon matched-pairs signed-rank) of the difference between amplitudes during PAG/DRN stimulation and control [bin size = 10 ms; interval of interest (IOI) = 0.140 to 0.640 s after stimulus for the forepaw and 0.250 to 0.750 s after stimulus for the hind paw]. (**B**) Box-and-whisker plots (median and quartiles) of inverted nociceptive-evoked FP response amplitude in the time point of maximum control response (left), the average *z* score within the IOI (middle), and the withdrawal rate (right) in fore- and hind paws (**P < 0.01; Wilcoxon matched-pairs signed-rank). (**C**) Linear regression analyses of the stability of the antinociceptive effects during continuous PAG/DRN stimulation (Imax, 50 Hz) for 1 hour on nociceptive-evoked FP amplitude (left), the average *z* score within the IOI (middle), and withdrawal rate (right) for the fore- and hind paws (*n* = 7 rats, four time points: immediately after initiation of PAG/DRN stimulation (0 min) and 15, 30, and 60 min after initiation of the PAG/DRN stimulation). Each dot represents the mean value on 16 nociceptive skin stimulations at a given time point in one rat (*P < 0.05; **P < 0.01; single linear regression using mean values). FP, FP voltage; −A, inverted amplitude; Avg. *z* score, averaged *z* score in the IOI; ns, not significant.

To clarify whether stimulation with the selected microelectrodes not only affects nociceptive motor behavior, we also assessed the effects on nociceptive-evoked activity in the hind and forepaw S1 cortices (*45*, *46*, *48*) in the same animals during weeks 4 to 5 PS. As shown in Fig. 3 (A and B), PAG/DRN stimulation practically abolished the nociceptive-evoked cortical FP and neuronal responses from both hind and forepaws. As expected from the initial tests, withdrawal reflexes were also strongly reduced in all animals (*n* = 8). Reduction of either the stimulation current (from Imax in steps of 10 μA while keeping stimulation frequency at 50 Hz) or stimulation frequency (20 and 5 Hz, while keeping current at Imax) lowered the analgesic efficacy (figs. S2 and S3). Henceforth, Imax and 50 Hz were kept throughout the remaining evaluation in each animal.

The inhibition of nociceptive responses was monitored at four time points during a 1-hour-long continuous stimulation to assess the stability of the antinociceptive effects of longer-lasting stimulation. Using single linear regression analysis, we found that the inhibition of cortical responses was unchanged (FPs in both cortical areas and average *z* score in the hind paw area) or somewhat reduced (by 31% for average *z* scores in forepaw cortical area, *P* < 0.05) and the inhibition of nociceptive withdrawal reflex rates in both fore- and hind paws was somewhat decreased (forepaw by 33%, *P* < 0.01 and hind paw by 29%, *P* < 0.01) over time (*n* = 7; Fig. 3C). While the reflex inhibition was still prominent at the 1-hour end point, it thus appears that the inhibitory effects on the spinal nociceptive withdrawal reflexes are less robust compared to the inhibitory effects on the nociceptive cortical input.

### Comparison with morphine-induced analgesia

**Fig. 4.**
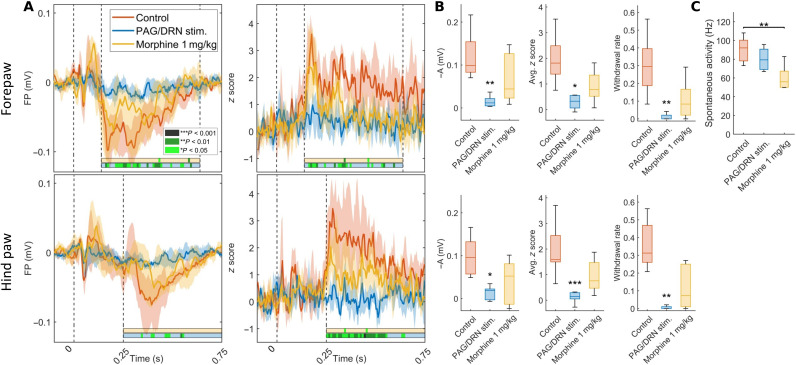
The effects of PAG/DRN stimulation and morphine on nociceptive-evoked FPs, neuronal responses, spontaneous neuronal activity in the S1 cortex, and nociceptive withdrawal reflexes. (**A**) Median of nociceptive-evoked FP amplitude (left) and neuronal responses *z* score (right) from contralateral fore- and hind paw S1 in three different conditions (*n* = 8 rats; color keys provided in topmost left inset). Graphics as in Fig. 3. Statistical comparisons were made with Friedman’s test with Dunn-Sidák post hoc. (**B**) Box-and-whisker plots (median and quartiles) represent the inverted nociceptive-evoked FP response amplitude in the time point of maximum control response (left), the average *z* score within the IOI (middle), and the withdrawal rate (right) in fore- and hind paws (*n* = 8; ***P < 0.001; **P < 0.01; *P < 0.05; Friedman’s test with Dunn-Sidák post hoc). (**C**) Box-and-whisker plots (median and quartiles) represent the spontaneous firing rate in S1 cortex at rest (*n* = 7; **P < 0.01, Friedman’s test with Dunn-Sidák post hoc).

We compared the effects of PAG/DRN stimulation with the analgesia induced by morphine (1 mg/kg, subcutaneously) in the same animals (*n* = 8) during weeks 4 to 5 PS to benchmark the analgesic efficacy of PAG/DRN cluster stimulation. The analgesia induced by morphine was less powerful than PAG/DRN stimulation (Fig. 4, A and B). Moreover, morphine caused obvious signs of behavioral sedation. For example, the rats usually appeared to fall asleep with slower breathing and closed eyes. Given the relatively modest analgesic effect of morphine found, we added 2 mg/kg subcutaneously about 30 min after the first dose to check that the morphine dose was not too low. However, there was no substantial additive analgesic effect (fig. S4). Morphine also depressed spontaneous neuronal activity in the S1 cortex, whereas signs of behavioral sedation or depression of cortical activity were not present during the PAG/DRN cluster stimulation (*n* = 7; Fig. 4C). These results indicate that PAG/DRN cluster stimulation yielded stronger analgesia with less severe adverse effects than morphine.

### Effects of PAG/DRN stimulation during hyperalgesia

**Fig. 5.**
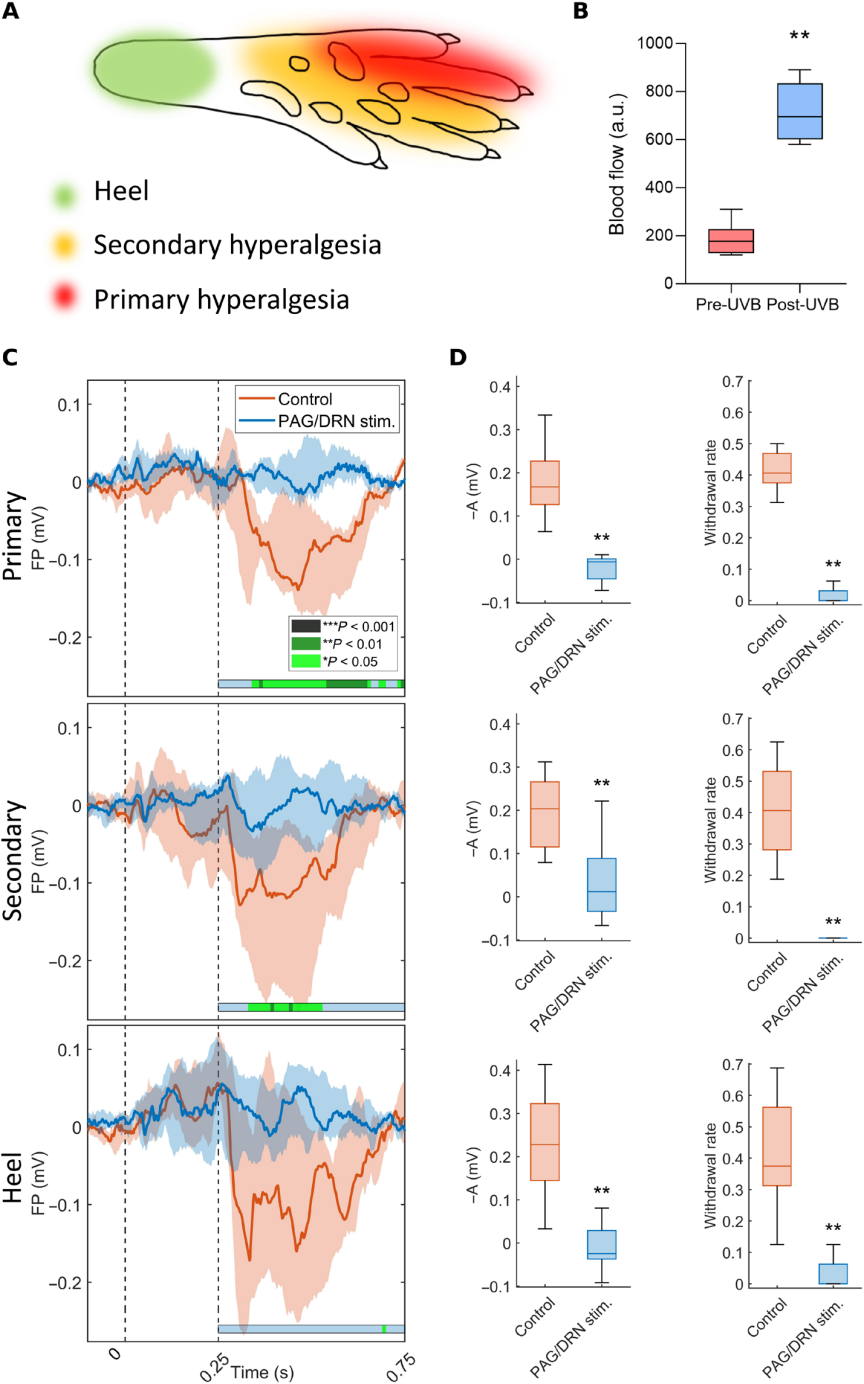
PAG/DRN stimulation abolishes nociceptive-evoked FPs and withdrawal reflexes also during hyperalgesia. (**A**) Illustration of the area directly irradiated with UVB light (primary hyperalgesia area, in red), the surrounding area (secondary hyperalgesia area, in yellow), and the heel (not affected by the irradiation, in green). (**B**) Box-and-whisker plots (median and quartiles) of blood flow before and 2 days after UVB irradiation (*n* = 8; **P < 0.005, Wilcoxon matched-pairs signed-rank test). a.u., arbitrary units. (**C**) Colored lines indicate the median of nociceptive-evoked FP amplitude across the animals (*n* = 8), in the control condition and during PAG/DRN stimulation, and in the hind paw’s primary, secondary, and heel area 2 days after UVB light induction. The shaded area around the median lines represents the interquartile range. The evoked responses are shown in the *x* axis from −0.1 to 0.750 s around the nociceptive stimulus onset. Statistical comparison between control-evoked FPs and during PAG/DRN represented by horizontal color-coded bars as in Fig. 3A. (**D**) Box-and-whisker plots (median and quartiles) represent the inverted nociceptive-evoked FP response amplitude in the time point of maximum control response (left) and the withdrawal rate (right) in the three different skin areas (*n* = 8; **P < 0.01; Wilcoxon matched-pairs signed-rank).

PAG/DRN stimulation was performed during ultraviolet B (UVB)–induced hyperalgesia (2 days after induction) during weeks 6 to 7 PS to clarify whether the PAG/DRN cluster stimulation can also block the nociceptive input to the S1 cortex in clinically relevant sensitized states. Three regions of interest were analyzed: primary hyperalgesic area (directly irradiated with UVB), secondary hyperalgesic area (immediately adjacent to the irradiated area), and heel (assumed to be outside the hyperalgesic area; Fig. 5, A and B). PAG/DRN stimulation strongly reduced nociceptive-evoked neuronal responses and FPs from the heel, primary, and secondary hyperalgesic areas (in all cases *P* < 0.01). The withdrawal reflex responses from all areas were also strongly reduced (*P* < 0.01; *n* = 8; Fig. 5, C and D, and fig. S5). These findings indicate that the antinociceptive effect of PAG/DRN stimulation is also powerful during hyperalgesia.

**Fig. 6.**
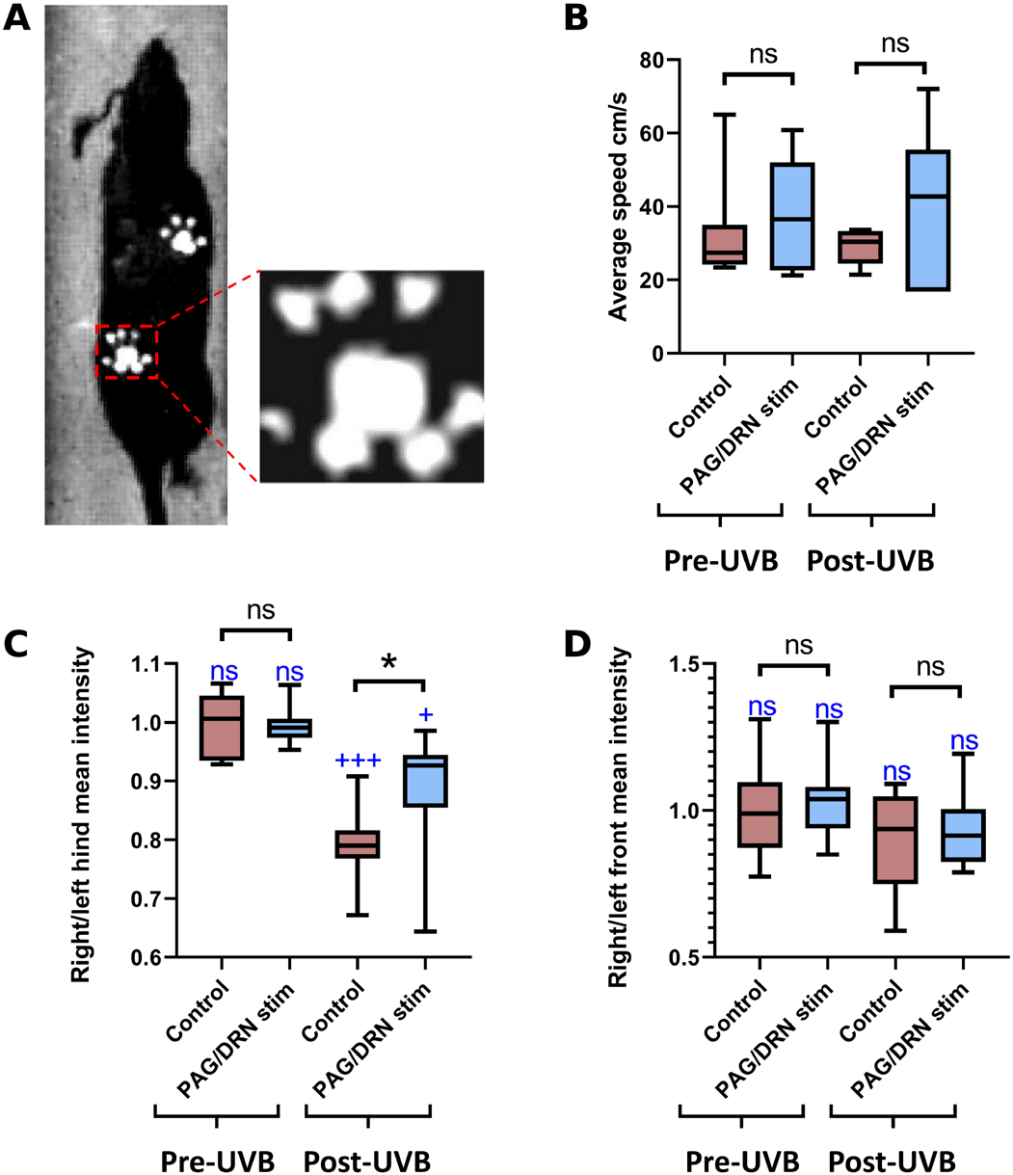
Effects of PAG/DRN stimulation on speed and paw print intensity during gait in normal conditions and hyperalgesia. (**A**) Example photo from a CatWalk video recording and a magnification of the right hind paw print. (**B** to **D**) Box-and-whisker plots (median and quartiles) represent the average speed (B) and the right/left mean intensity ratio of the paw print intensity of the hind paw (C) and forepaw (D) in the two different conditions before and after UVB irradiation as indicated below each plot [*n* = 7; *P < 0.05, ns (in black), Wilcoxon matched-pairs signed-rank test; ns (in blue), +++*P* < 0.001, +*P* < 0.05, one-sample *t* test].

An assessment of gait (Fig. 6A) was performed during weeks 6 to 7 PS to evaluate whether PAG/DRN cluster stimulation affects normal gait and gait modified by hyperalgesia. Before UVB irradiation, the right/left ratios of the mean intensity of the forepaw and hind paw prints, with or without PAG/DRN stimulation, were normally distributed around 1 (i.e., symmetrical gait). Thus, the ratios were not affected by PAG/DRN stimulation in control conditions. Although the gait speed tended to be increased during PAG/DRN stimulation, this potential effect did not reach statistical significance, indicating no impairment of normal gait (Fig. 6, B to D). Two days after UVB irradiation, the mean intensity of the inflamed hind paw print was, for all seven animals, lower as compared to the left hind paw (correspondingly, the right/left ratio differed significantly from a normal distribution around 1, *P* < 0.001) indicating asymmetric gait (Fig. 6C and table S1). Under this condition, PAG/DRN cluster stimulation significantly reduced the asymmetric hind paw prints (right/left ratio closer to 1) during gate but did not affect the forepaw right/left ratio. Again, the gait speed tended to increase, but this did not reach statistical significance (*n* = 7; Fig. 6, B and C).

### Reliability of cluster stimulation effects

**Fig. 7.**
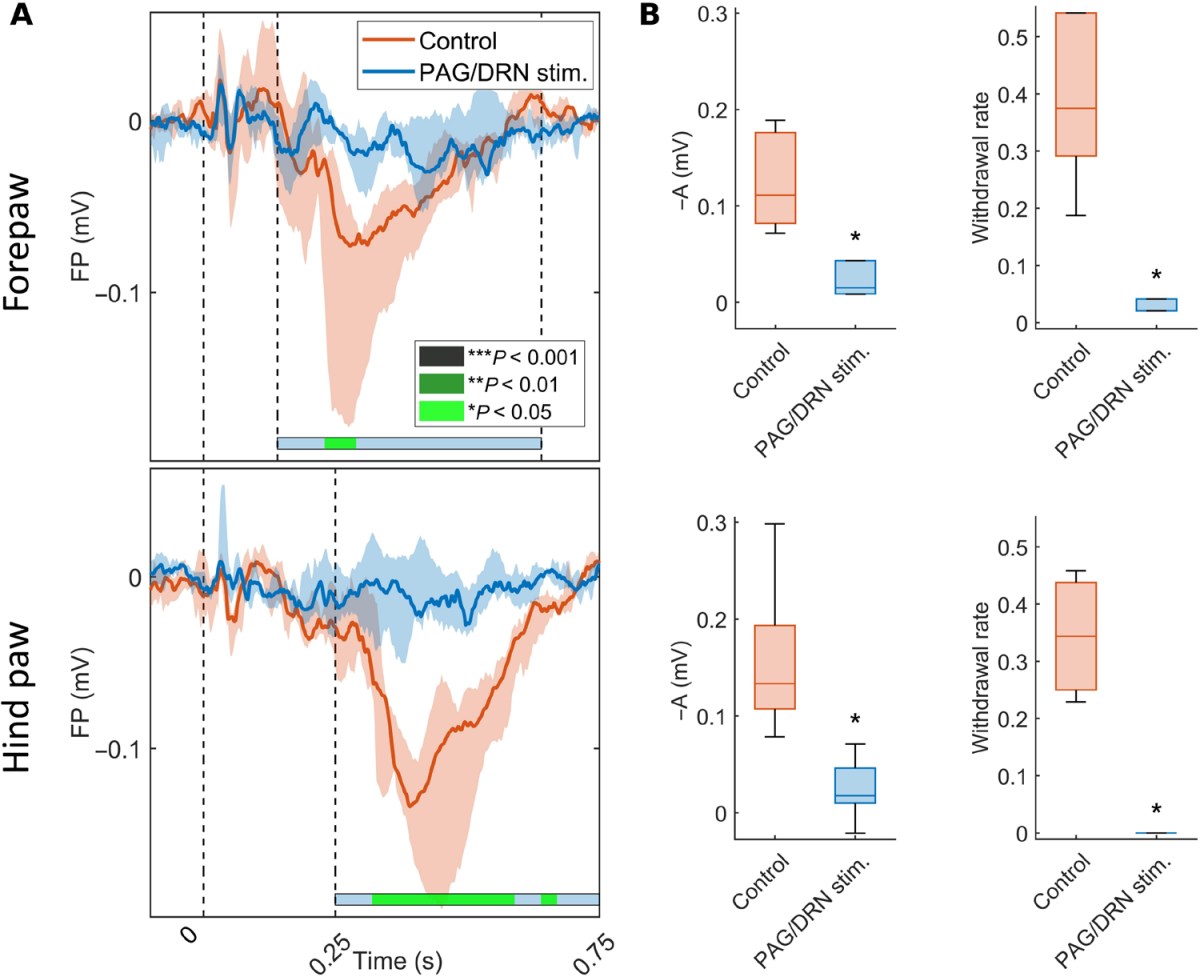
Analgesic effects induced by PAG/DRN stimulation are still powerful in the long-term follow-up. Effects of PAG/DRN stimulation on cortically evoked nociceptive FPs and withdrawal rate during weeks 10 to 11 PS using the same stimulation configuration and parameters as set during weeks 2 to 3. (**A**) Colored lines indicate the median of nociceptive-evoked FP amplitude across the animals (*n* = 6), recorded in contralateral fore- and hind paw S1 in the control condition and during PAG/DRN stimulation. The shaded area around the median lines represents the interquartile range. The evoked FP responses are shown in the *x* axis from −0.1 to 0.750 s around the nociceptive stimulus onset. Statistical comparison (Wilcoxon matched-pairs signed-rank) between control nociceptive-evoked FPs and during PAG/DRN stimulation represented by color-coded bars as in Fig. 3A. (**B**) Box-and-whisker plots (median and quartiles) represent the inverted nociceptive-evoked FP response amplitude recorded in contralateral fore- and hind paw S1 in the time point of maximum control response (left) and the withdrawal rate (right) in fore- and hind paws (*n* = 6; *P < 0.05; Wilcoxon matched-pairs signed-rank). FP, field potential voltage; -A, inverted response amplitude.

PAG/DRN stimulation was retested during weeks 10 to 11 PS using the stimulation parameters set in the selection phase (weeks 2 to 3 PS) to clarify the reliability of the described analgesic effects. Again, the PAG/DRN cluster stimulation caused a powerful inhibition of the cortical signals (both nociceptive-evoked FP and neuronal responses) and the motor reflexes (*n* = 6; Fig. 7 and fig. S6). In addition, the microelectrode impedance was unaltered (fig. S7A). Together, these and the preceding findings indicate that reliable, stimulation-produced analgesia was achieved throughout the study.

### Effects of PAG/DRN stimulation on tactile-evoked potentials

**Fig. 8.**
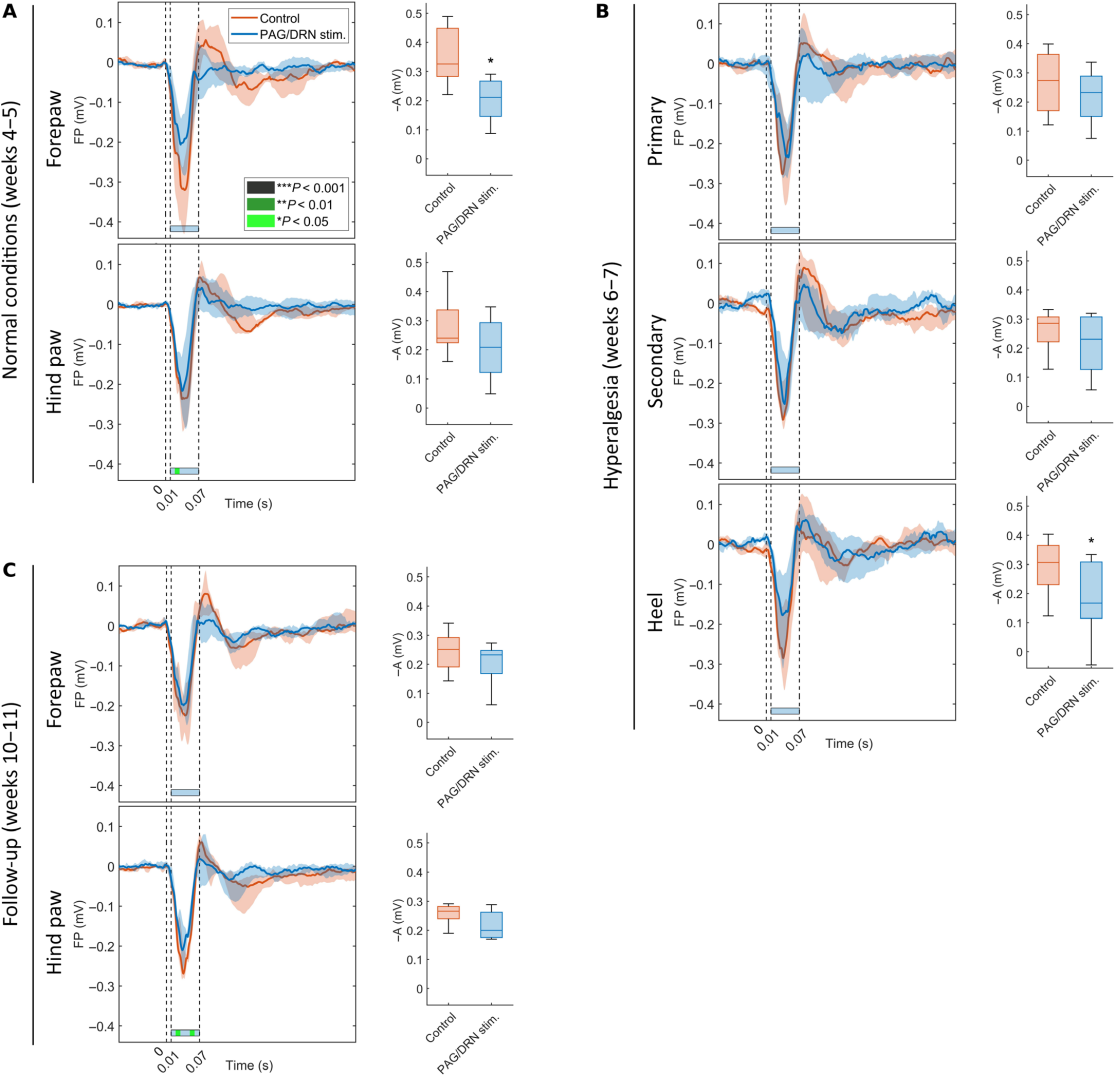
Minor effects of PAG/DRN stimulation on tactile-evoked cortical signals and withdrawal reflexes in control conditions and hyperalgesia. The median and interquartile range of tactile-evoked FP amplitude in the left panel of (**A**) to (**C**) throughout the study during the normal condition in fore- and hind paws weeks 4 to 5 [(A); *n* = 8], during hyperalgesia weeks 6 to 7 [(B); *n* = 8, hind paw] and weeks 10 to 11 [(C); *n* = 6]. Time axis from −0.1 to 0.4 s with respect to stimulus onset. The horizontal bars under each graph provide the statistical significance of the difference between PAG/DRN stimulation and the control (bin size = 10 ms, IOI = 0.01 to 0.07 s after stimulus represented by the second and the third vertical dashed lines, Wilcoxon matched-pairs signed-rank). Box-and-whisker plots (median and quartiles) in the right panels of respective (A) to (C) represent the inverted nociceptive-evoked FP response amplitude in the time point of maximum control response in fore- and hind paws or primary, secondary, or heel area during hyperalgesia (*P < 0.05, Wilcoxon matched-pairs signed-rank).

Recordings of tactile-evoked FP and neuronal responses during PAG/DRN stimulation were compared to control to clarify whether the PAG/DRN stimulation causes a general insensitivity to cutaneous sensory input or has a selective analgesic effect. Relatively minor (in a minority of cases reaching a significance of *P* < 0.05; Fig. 8 and fig. S8) effects were found on tactile-evoked cortical FP and neuronal responses in normal conditions and during hyperalgesia. This observation is in sharp contrast to the powerful inhibition of nociceptive cortical signals indicating a preferential effect of PAG/DRN cluster stimulation on nociception.

## DISCUSSION

This study was motivated by the fact that patients with debilitating intractable pain often lack effective analgesic treatments free of adverse effects interfering with daily activities and negatively influencing the quality of life. To harness the power of the complex endogenous analgesic systems known to exist in PAG/DRN but proven to be difficult to use clinically, we developed an implantable cluster of ultrathin microelectrodes allowing fine-tuned granular stimulation in deep targets. We combined conventional assessments of both nociceptive withdrawal reflexes and behavioral alterations in awake, freely moving animals with simultaneous recordings of nociceptive-evoked activity in a cortical area associated with the perception of pain in humans to improve the validity of the study (*46*, *49*, *50*). We show that it is possible to provide reliable and powerful analgesia during normal conditions and UVB-induced hyperalgesia [a translational hyperalgesia model (*51*)] without noticeable sedation or other adverse side effects by stimulating an individualized selection of microelectrodes implanted in the caudal PAG/DRN. We found no impairment of normal motor performance (gait) and only small effects on the tactile cortical responses and spontaneous neuronal activity at rest, indicating that highly selective analgesia was accomplished. By contrast, morphine, used for benchmarking, did not match the analgesic effects seen during 3D microelectrode cluster stimulation of the PAG/DRN. Besides, the present data, showing minimal behavioral effects during PAG/DRN when using a selected subgroup of microelectrodes, are consistent with the notion that part of the PAG/DRN is involved in preventing nociceptive stimuli from compromising animals’ ability to cope with external threats (*52*).

### Animal model

Previous studies have shown that nociceptive-evoked activity in the awake rodent primary somatosensory cortex mimics essential aspects of human pain, including sensory discriminative aspects (*46*, *47*, *50*, *53*) and typical features of persistent pain such as sensitization and primary and secondary hyperalgesia. Recordings of nociceptive-evoked activity in the awake rodent primary somatosensory cortex therefore provide a powerful translational animal model of pain (*45*). On the other hand, while proving useful in the initial selection of microelectrodes in the present study and being the most widely used animal model of pain (*54*), nociceptive withdrawal reflexes show many discrepancies to the perception of pain (*45*, *55*). For example, secondary hyperalgesia, which is dependent on central mechanisms, is not mimicked by increased withdrawal reflexes (*45*). Conversely, reduced or altered withdrawal reflexes may be due to motoneuronal inhibition (*56*).

Moreover, other areas of PAG than those stimulated here are known to produce “freezing” behavior (*6*, *10*, *57*), which may be mistaken for analgesia if only nociceptive withdrawal reflexes are monitored. To improve validity of the animal study, we therefore inferred the presence of analgesia during stimulation of PAG/DRN from both inhibited nociceptive-evoked intracortical activity and from inhibited nociceptive withdrawal reflex responses in the absence of noticeable behavioral effects or impaired gait.

Despite an almost total blockage of nociceptive withdrawal reflexes, only a partial reversal of the hyperalgesia-induced motor disturbance was found during PAG/DRN stimulation. This result may suggest that plastic changes in the motor control, known to be induced by nociceptive stimuli in, for example, cerebellar circuits (*58*–*60*), are not immediately erased by PAG/DRN stimulation.

### Analgesic power and sedation

Comparing the PAG/DRN stimulation to the effects of morphine, which is still widely used and one of the strongest analgesics known (*61*), reveals a superior analgesic effect of the 3D microelectrode cluster stimulation even when compared to morphine at high doses. The comparison also highlights the absence of evident sedation or impairment of motor performance during 3D cluster stimulation. Sedation is a ubiquitous and highly problematic side effect of centrally acting analgesics, which interferes severely with mental abilities and normal life (*62*–*64*).

### Biocompatible technique and stimulation specificity

The developed method for PAG/DRN stimulation uses a gelatin-embedded cluster of highly flexible and ultrathin microelectrodes, each equipped with a silicon blob distally to reduce the risk for tissue damage, that is gently spread out during insertion into the PAG/DNR area. Gelatin provides the necessary structural support, and by allowing it to expand during the initial phase of the insertion, the embedded microelectrodes are spread before individually penetrating the target. The stability of effects produced by the same microelectrode subset and stimulation parameters, together with the low stimulation current per microelectrode needed (median, 30 to 40 μA for nearly complete inhibition of cortical responses) during the 11 weeks tested, indicates high positional stability of the implanted microelectrodes. These findings are consistent with previously found high biocompatibility of both gelatin and ultrathin microelectrodes (*37*, *42*–*44*, *65*, *66*).

An appropriate subgroup of microelectrodes was selected in each animal to reach a high stimulation specificity based on the effects on nociceptive withdrawal reflexes and the absence of adverse behavior. During the selection phase, it became clear that many microelectrode sites in PAG/DRN caused adverse effects, despite being located in the region of PAG/DRN, in which stimulation-induced analgesia has been reported (*6*, *10*, *67*). These findings are in agreement with previous microelectrode mapping studies reporting difficulties to localize purely analgesic regions in PAG/DRN devoid of adverse effects and support the view that the organization of this region is very complex (*7*, *68*) and compose different microcircuits of, e.g., GABA and glutamate neurons with divergent end targets that can produce competing behaviors (*20*).

In all animals, the selected combination of 3 to 5 microelectrodes (of at least 15 microelectrodes) had active sites in more than one plane (interplane distance between contacts: 300 μm), whereas many microelectrode sites in the same planes and regions produced behaviorally adverse effects. Moreover, with no exception, adverse effects were also elicited when stimulating the selected subgroup of microelectrodes with higher stimulation current or frequency than used during the evaluations, indicating proximity to neuronal networks serving other functions. Notably, powerful and widespread analgesia without adverse effects could only be achieved by combined stimulation of a selected subgroup of the implanted microelectrodes with separated sites in 3D (Fig. 2B). Using a combination of microelectrodes also appears to enable reduction of the current ejected from each microelectrode, in turn reducing current spread around each microelectrode. Thus, the precise location of the multiple stimulation sites is critical to enable activation of key components of the analgesic circuits in an individualized fashion. Conventional DBS probes cannot provide this fine-tuned granular stimulation pattern in 3D. These electrodes use contacts in the millimeter scale on a common carrier body (*69*, *70*). Instead of a granular stimulation pattern with multiple “hot spots”, conventional DBS probes provide continuous stimulation fields fading in intensity with distance. Hence, the restricted possibilities to adapt the stimulation field to a local and potentially complex neuronal network organization may contribute to the unreliable therapeutic effects of conventional DBS.

The developed 3D cluster design for neural stimulation can be tailored to brain regions with different architecture. For example, the number and 3D arrangement of microelectrodes and microelectrode contacts in the gelatin vehicle and stimulation parameters can be easily modified. The principle of selecting the therapeutically effective microelectrodes devoid of side effects from an implanted cluster is an uncomplicated method of fine-tuning the stimulation pattern, thereby adapting to inevitable variations in microelectrode placements during surgery and age- or disease-dependent structural changes in the brain (*71*, *72*). Hence, the improved stimulation specificity offered by the 3D cluster design and accompanying selection method has the potential of being useful for treatments of a variety of neurological and psychiatric conditions.

### Limitations of the study

While different stimulation parameters (number of microelectrodes, frequency, and current) were tested to narrow down a therapeutically useful window, further optimization is possible. For example, stimulation frequencies other than those tested could provide equal or superior effects. The incentive for further optimization would be to reduce the risk for side effects and excessive energy expenditure and reduce metabolic load on the stimulated neurons. Such optimization may also mitigate the partial decrease in inhibition of withdrawal reflexes found over the 1-hour stimulation period. Notably, once the stimulation parameters were set after the initial 2 to 3 weeks, we kept the same combination of microelectrodes and stimulation parameters to compare the analgesic efficacy until weeks 10 to 11.

Although the animals studied did not show any noticeable side effects during PAG/DRN stimulation using the selected group of microelectrodes, we cannot exclude the induction of more subtle side effects that are not accompanied by observable behavioral changes and not accompanied by changes in spontaneous cortical (S1) activity. To bring this technique to the clinic, an upscaling in length and number of microelectrodes and necessary safety studies will be the next steps.

## MATERIALS AND METHODS

### Study design

This study applied a novel probe design for electrical brain stimulation in the PAG/DRN areas. We evaluated its effects on nociception, tactile sensation, and behavior in awake, freely moving rats. To address this research objective, we performed controlled laboratory experiments in which we examined therapeutic and adverse effects induced by PAG/DRN stimulation by (i) observation of behavior, (ii) computerized analysis of nociceptive- and tactile-evoked intracortical signals in the contralateral forelimb and hindlimb S1 cortex, (iii) quantification of withdrawal reflexes in response to nociceptive stimuli, and (iv) computerized quantification of changes in gait (speed and paw print intensity). The sample size was defined by previous experimental experience, and the exact number for each experiment is indicated in the figure legends. Exclusion criteria were applied as described in the dedicated section. In the hyperalgesic state, the assessments of withdrawal rate and recordings of cortical activity were performed once per condition (16 nociceptive/tactile stimulations). During a 1-hour continuous PAG/DRN stimulation, the assessment was performed four times (16 nociceptive stimulations per time point). In the rest of the experiments, the assessment was performed three times per condition (48 nociceptive/tactile stimulations). Rat gait measurements were performed before the induction of hyperalgesia (with and without PAG/DRN stimulation, five runs per animal and condition) and 2 days after the induction of hyperalgesia (with and without PAG/DRN stimulation, five runs per animal and condition). The experimenters were not blinded to the intervention. The analysis was fully computerized to minimize the risk of bias.

### Probe manufacturing

#### Microelectrodes for stimulation

Microelectrode clusters were manufactured from 16 platinum-iridium (PtIr)–annealed microwires (diameter, 12.7 μm; 90% Pt and 10% Ir; California Fine Wire, USA), which were coated with a 4-μm parylene C insulating layer in a Compact Bench Top Coating System (LabTop 3000, Para Tech Coating Inc., CA, USA). On each wire, the insulation was removed for 300 μm at one of three different levels, counting from the tip: 0 to 300, 300 to 600, and 600 to 900 μm in a Laser Machining System (LaserMill 50, standard micromilling system, New Wave Research Inc., USA) using focused UV radiation (wavelength, 355 nm) under a 20× objective and cut to the appropriate length in both ends in the same micromilling system (fig. S9). A 30- to 50-μm-diameter silicon droplet (NuSil Technology, MED1-4213, USA) was manually added at the distal end to each wire under a stereomicroscope (Olympus, SZX16, Japan) to avoid sharp damaging contours at the tip of the electrode. The distal part of the wires was kept at −78°C and then dipped in room temperature (RT) 30% gelatin 289 Bloom strength (Gelita, Medella Pro 1500, USA), dissolved in Milli-Q water for a length of 1.5 mm using a dipping machine (Holmarc Opto-Mechatronics, Dip Coating Unit, HO-TH-01, India), and left to dry at RT for at least 1 hour. The wires were also deinsulated for ~3 mm on the proximal end (to the cable connector) with a butane flame (Portasol Pro Piezo 75, USA) to enable soldering to the cable connector. The distal parts of five to six wires were aligned and arranged on an antiadherent surface under a stereomicroscope (Zeiss, Stemi Dv4, USA), cooled to −78°C in dry ice, and sprayed with a solution of 10% gelatin (dissolved in Milli-Q water at 50°C; 101 Bloom strength, Gelita, Medella Pro 1500, USA) to freeze-fixate them in a thin sheet of gelatin, which was then left to dry a minimum of 1 hour. The gelatin sheet was then released from the antiadherent surface. Three sheets composed of five, six, and five wires, respectively, with a specific arrangement (with respect to where insulation had been removed relative to the distal end), were stacked and aligned under a stereomicroscope, trimmed with a knife, and placed in a plexiglass mold (Prototech AB, Helsingborg) with 9-mm-long and 350-μm-diameter cylindrical space and a cone-shaped distal tip. A sonicated (free of air bubbles) solution with 30% gelatin (dissolved in Milli-Q water at 50°C; 101 Bloom strength) was injected into the mold and subsequently dried overnight in a humidity- and temperature-controlled chamber at 50% humidity and 21°C (Rcom King Suro Max-20 digital incubator). The proximal parts of the wires were pretinned (Solder Chemistry sc 170, Germany) and soldered to a male Omnetics connector (Plexon, CON/32m-VA8828-001, USA). A soldering iron attached to a micromanipulator (Luigs & Neumann, Feinmechanik und Elektrotechnik GmbH, Germany) and a stereomicroscope (Olympus, SZX7 0.5-4X, Japan) were used for precise soldering. A 25-μm pure platinum wire (Goodfellow Cambridge Ltd., PT005114, UK) was soldered to the connector on the channel dedicated to the ground. The soldered parts of the connector and the deinsulated proximal parts of the wires were covered with medical grade epoxy (Epoxy Technology, EPO-TEK OG198-54 and 55, USA) and cured with UVB light (Honle, bluepoint LED eco). Last, when dry, the probe was released from the mold. Only probes with a straight shape were used. Before surgery, each probe was quickly dipped in a 4°C saline solution. The drop in impedance between the ground wire and microelectrodes was measured (nanoZ, White Matter LCC, nanoZ v1.4.0 software, USA) to verify the electrical contact between each microelectrode and the connector. Probes with less than 15 connected microelectrodes were excluded.

#### Microelectrodes for recordings

The same type of parylene C–insulated PtIr wires used for manufacturing stimulation microelectrodes, but deinsulated by 50 μm, were used for intracortical neuronal recordings. The recording probe consisted of two wire bundles adapted to intracortical recordings in the forepaw and hind paw primary somatosensory cortex, respectively, wherein each bundle comprised three PtIr wires embedded in gelatin. The level of the deinsulated region differed for the three wires in each bundle, corresponding to a cortical depth of ~1400, ~1000, or ~ 750 μm, respectively, from the brain surface. The deinsulation, silicon droplet addition to the microelectrode tips, and dip coating in gelatin were made according to the same procedure described for the stimulation microelectrodes (see above). Two parallel bundles of 12.7-μm wires separated by 1.6 mm were glued together with a drop of epoxy at ~3 mm from the microelectrode tip (Epoxy Technology, EPO-TEK OG198-54 and 55, USA) that was cured with UVB light (Honle, bluepoint LED eco, Germany). The proximal end of each respective microelectrode and the ground wire was soldered to a male Omnetics connector (Plexon, CON/8o25m-10PA8393-001, USA). Afterward, the soldered area and the proximal part of the wires were insulated with epoxy.

### Animals and surgical procedures

Approval for the experiments was given in advance by the Malmö/Lund Animal Ethics Committee on Animal Experiments (ethical permit M4480-18). The animals had free access to food (except during CatWalk sessions, see below) and water. They were kept in a 12-hour light/dark cycle at a controlled environmental temperature of 21°C and 65% humidity. Ten female Sprague Dawley rats were anesthetized with isoflurane 2% (Isobavet, Apoteksbolaget, Sweden) with 40% oxygen and 60% nitrous oxide and then kept at 1.2 to 2% isoflurane, delivered through a nose mask (David Kopf Instruments, CA, USA). After being shaved, the head was fixed in a stereotactic frame (Neurostar, Robot Stereotaxic instrument, Germany). The rats were kept on a heated table at 37°C, and the implantation was performed with a programmed micromanipulator (Neurostar, StereoDrive 4.0.0, Germany). After subcutaneous injection of 0.5 ml of local anesthetic [a mixture of xylocaine (2 mg/ml) and adrenalin (1.25 μg/ml); Dentsply Ltd., Surrey, UK] in the surgical area, a midline incision was performed to expose the skull.

After removal of connective tissue, the head position was measured and calibrated with the micromanipulator software. Four stainless-steel anchor screws (Agnots, MCS1x2, Sweden) were screwed into the skull. A ~2.5 mm by 1.5 mm craniotomy on the left side was manually drilled (Kopf Model 1474 High-Speed Stereotaxic Drill, USA) around the coordinates for the recording microelectrodes [−0.84 mm rostral, −2.4 mm lateral, and −1.45 mm to bregma for the hindlimb area; and −0.84 mm lateral, −4 mm lateral, and −1.45 mm ventral to bregma for the forelimb area (*45*, *48*)]. An additional hole on the right side, about 1.5 mm by 1.5 mm, was drilled around the coordinates to insert the stimulation probe (−8 mm rostral, 0 mm lateral, and −6.2 mm ventral to bregma). The brain surface was exposed, and the dura mater and the pia mater were cut to reduce dimpling of the brain tissue and possible compression damage or displacement of the microelectrodes. The stimulation probe was mounted on a holder and inserted into the brain, with a 30° angle to cross the midline, in two speed-steps: 1000 μm/s for initial penetration to a pretarget region 2 mm from the target and then 100 μm/s to target. A pause of 3 min between the two insertion steps was made to allow the gelatin to expand and the embedded wires to separate. Dental cement (RelyX Unicem Self-Adhesive Universal Resin Cement) was used to anchor the probe to the skull and the screws. Warm saline was used to dissolve the gelatin on the external part of the probe before securing the stimulation probe connector in dental cement. The ground wire was inserted in a drilled hole on top of the brain in contact with the dura and fixed with dental cement. The array of recording microelectrodes was inserted into the brain with two speed-steps (1000 μm/s for the first 1000 μm and 100 μm/s for 450 μm) using a micromanipulator (David Kopf Instruments, USA). The recording microelectrode array and contact were then fixated to the skull with dental cement. The ground wire was inserted through a drilled hole on top of the brain surface and secured with dental cement.

During surgery, the eyes and the exposed brain area were constantly kept moist with 0.9% saline solution, and 5 ml of saline was injected subcutaneously to avoid dehydration. About 20 min before the end of the surgery, the rats received subcutaneous injections of Temgesic (0.01 mg/kg; Buprenorfi, Schering-Plough, Belgium) for postoperative pain relief, and they were carefully monitored during awakening.

### Verification of stimulation probe location

CT imaging and the superimposition of the obtained 3D images with the Waxholm brain atlas (*73*) were performed in vivo as soon as possible after the probe implantation to verify the probe location within the PAG/DRN area. The rats were anesthetized with 1 to 2% isoflurane with 40% oxygen and 60% nitrous oxide, and 3D CT imaging was performed with a nanoPET/CT machine (Mediso, nanoScan PET/CT at Lund University Bioimaging Center). A sensor (Small Animal Instruments Inc., USA) was placed under the anesthetized animals to monitor the breathing during imaging. Circular scans were made [x-ray energy, 65 kilovolt peak (kVp) and exposure time, 1300 ms], reconstructed in 3D, and exported in DICOM (Digital Imaging and Communications in Medicine) format to measure the spread of microelectrodes. They were then opened in ITK-SNAP to estimate the diameter of the distal part of the cluster, which was found to be about 0.7 mm. Helical scans (x-ray energy, 65 kVp and exposure time, 1300 ms) were taken to determine the stimulation probe location within the skull. The images were 3D reconstructed and exported in DICOM format. ITK-SNAP (*74*) was used to align the CT scan 3D images with the rat Waxholm brain atlas to verify the location of the stimulation probe within the PAG/DRN (fig. S1A). After aligning the images, the locations of the stimulation probe tip and the point where the probe entered the skull were identified in the atlas coordinate system. For each animal, the spatial domain of tissue within the atlas containing the deinsulated electrode sites was approximated as a cylinder with a height of 0.9 mm and a diameter of 0.7 mm, starting at the tip location and pointing to the entering point at the scull. This spatial domain for all rats was then plotted together with the spatial extent of the PAG given by the atlas to facilitate the visual assessment of probe location with respect to the PAG (fig. S1B and movie S1). In one rat, it was not possible to perform CT imaging because of the detachment of the contacts from the skull. In this case, the probe placement was instead verified through cresyl violet staining (more details can be found in fig. S1C).

### Skin stimulation and reflex responses

Nociceptive heat stimulation of the skin was made with a CO_2_ laser pulse (wavelength, 10.6 μm; MedArt VariMed Diode Laser System, Denmark); pulse duration 20 to 32 ms, beam diameter 3.0 mm, and 5-W output power (*75*, *76*). Tactile stimulation was delivered through a handheld magnetic tactile stimulator (with onset time <2 ms from initiation). The laser and the tactile stimulator were connected to a Master 8 stimulator (AMPI, Jerusalem, Israel) to trigger the events and the Plexon data acquisition system (Plexon Inc., USA) to record event time stamps synchrony with intracortical recordings.

Before every experimental session (each lasting a maximum of 4 to 5 hours with 24 hours minimum pause in between), nociceptive withdrawal reflex thresholds were determined using CO_2_ laser stimulation for each of the four paws during the microelectrode selection experiments (weeks 2 to 3 PS), and in the contralateral paws to the S1 recording probe in the following experiments. The CO_2_ laser thresholds to elicit a withdrawal reflex response were defined as the pulse duration eliciting three responses out of the five laser stimulations. This pulse duration was then used in the following tests in the session, except for the microelectrode selection experiments in which 2 ms above the threshold was used. A maximum of 32-ms pulse duration was used to avoid damage to the rats’ skin, and the reflex response to each stimulation was noted in all the experiments.

### Induction of local hyperalgesia

The rats were briefly anesthetized with isoflurane 2% with 40% oxygen and 60% nitrous oxide. The distal lateral plantar part of the right hind paw area (contralateral to S1 probe) was irradiated with UVB light (Fig. 4A), while the rest of the paw was covered with a UVB opaque material. The UVB source consisted of a set of four TL/01 fluorescent tubes (between λ = 305 to 315 nm, *k*_max_ = 311 nm, double pins spaced 2.8 cm; Phillips, UK), producing an even field of irradiation. The UVB lamp was calibrated before each experiment using a Variocontrol meter (Herbert Waldmann GmbH & Co. KG, Germany). The UVB lamp was positioned 3 cm above the hindlimb. The UVB dose was adjusted for each rat to be 1.3 mJ/cm^2^, an intensity known to produce robust hyperalgesia but below the blistering threshold (*45*). A laser Doppler flow meter (Moor-LAB, Moor Instruments, UK) was used to measure the skin blood flow before and 2 days after irradiation in all rats, confirming robust induction of skin inflammation (Fig. 5B).

### Procedures for initiating electrical stimulation and data acquisition

Electrophysiological recordings and PAG/DRN stimulation were performed with the rats placed on a metal grid surface enclosed by a 20 cm by 25 cm plexiglass cage. The rats were briefly anesthetized with isoflurane 2% with 40% oxygen and 60% nitrous oxide to connect the implanted probes to the electrical stimulation and recording system cables. They were then allowed to habituate to the experimental setup at least 20 min before the start of each experimental session. The impedance of the recording and stimulation microelectrodes was measured (nanoZ, White Matter LCC, nanoZ v1.4.0 software, USA; fig. S7, A and B). Then, the cortical neural probe was connected via a headstage (gain 1), a commutator, and a preamplifier (gain 1000) to a Plexon multichannel neural data acquisition system for recordings of evoked FPs and neuronal responses and spontaneous firing rate at a sampling rate of 1 and 40 kHz, respectively. An in-house developed MATLAB software package (MATLAB and Statistics Toolbox, The MathWorks Inc., Natick, MA, USA) analyzed impedance measurements, stimulation control, and data collection related to stimulation. The stimulation probe was connected to a current-controlled stimulator (Plexon Inc., PlexStim v2.2, USA) via a commutator (Plexon Inc., USA). The stimulation was delivered at a frequency of 50 Hz (except in experiments involving analysis of effects of stimulation frequency) and with biphasic charge-balanced squared pulses (2 × 50-μs negative square pulse followed by a positive square pulse). Stimulation was always made against a ground electrode placed in contact with the brain.

### Selection of microelectrodes and Imax for PAG/DRN stimulation

To identify the microelectrodes in PAG/DRN that can elicit antinociception upon stimulation, the effect of each microelectrode was tested on CO_2_ laser–evoked withdrawal reflex responses in all four paws during weeks 2 to 3 PS. The CO_2_ laser pulse duration was kept at 2 ms above the reflex thresholds to ensure a consistent withdrawal reflex in response to all stimuli. For each paw, 10 CO_2_ laser pulses were delivered with an interval of at least 2 to 3 s, randomly shifting the skin site between stimulation pulses to reduce the risk of sensitization of the skin. All microelectrodes through which stimulation yielded a block of all nociceptive withdrawal reflexes in at least one of the paws at an intensity of a maximum of 50 μA were considered potentially analgesic. Electrodes that proved to elicit observable side effects were noted. The proportion of single microelectrodes showing potential analgesia, side effects, or no effects was calculated from the last performed experimental session in each rat. Subsequently, on the basis of this initial screening, various combinations of microelectrodes (mean number of combinations tested 29.75, SD 16.74) were tested to find a subset of microelectrodes and a current (30 to 50 μA defined as Imax), yielding complete forelimb and hindlimb reflex analgesia without behavioral abnormalities. Two to three sessions were usually needed to find these parameters. The selected microelectrode combination and Imax found for each rat at this stage were used in all subsequent sessions.

### Assessment of cortical antinociceptive effects of different stimulation frequencies and intensities

Cortical recordings were made during nociceptive stimulation at the reflex threshold of the right forepaw and right hind paw (16 stimulations of each paw) in the control condition and during 50-, 20-, or 5-Hz PAG/DRN stimulation (at Imax). This procedure was performed three times for each condition in each animal. Stimulation frequencies of 90 and 130 Hz were attempted in all rats but discontinued because they always induced adverse behavioral effects (such as alertness and escape behaviors). The impact of stimulation current was assessed in a separate session while keeping the frequency of PAG/DRN stimulation constant (50 Hz). The procedure was performed three times during the control condition and during PAG/DRN stimulation (50 Hz) at Imax and 10 and 20 μA below Imax.

### Assessment of durability of PAG/DRN stimulation–induced analgesia

Cortical recordings were made during 16 nociceptive stimulations, at the reflex threshold, of the contralateral fore- and hind paws, during the control condition and four times during a 1-hour long PAG/DRN stimulation (at Imax and 50 Hz) at 0, 15, 30 and 60 min from the onset.

### Assessment of reliability of PAG/DRN stimulation–induced analgesia

PAG/DRN stimulation was repeated during weeks 10 to 11 PS using the same PAG/DRN stimulation parameters in each animal as a set during the selection phase and the same skin stimulation protocol as described above to assess the reliability of stimulation effects over time.

### PAG/DRN stimulation during UVB-induced skin inflammation

The contralateral hindlimb was divided into three different areas of interest: primary hyperalgesia (where the hind paw was directly exposed during the UVB treatment), secondary hyperalgesia (the area nearby the primary hyperalgesia area), and heel (presumed to be unaffected by the hyperalgesia; Fig. 5A). Withdrawal thresholds for CO_2_ laser pulses were gauged in the three regions of interest in each session. Recordings in the S1 cortex were performed during the delivery of 16 nociceptive and 16 tactile stimuli with or without PAG/DRN stimulation 2 days after UVB irradiation.

### CatWalk training and experimental procedures

The rats were food-deprived 16 to 20 hours before each training (taking place during 5 to 7 days) that involved running along a 1.5-m-long CatWalk XT 9.1 (Noldus Information Technology, The Netherlands) reach a small piece of pellet at the end of the CatWalk. After the training period, a pre-UVB session, consisting of five runs per animal, was performed in the control condition and during PAG/DRN stimulation. A 40 cm by 10 cm area along the CatWalk was video-recorded from underneath, the paws in contact with the glass plate causing dispersion of light, making the paw prints easily detectable and quantifiable in the video recording. Two days following UVB irradiation, the procedure was repeated with the same experimental settings during the control condition and PAG/DRN stimulation. Mean paw print intensity was calculated for each run through the Noldus data acquisition system and exported in an Excel file for further analysis. Paw prints were classified automatically by the CatWalk software and were validated visually.

### Comparison of analgesic effects of PAG/DRN stimulation and morphine treatments

Withdrawal thresholds were taken for the fore- and hind paws, contralateral to the S1 recording microelectrodes. S1 cortical recordings were made during 16 consecutive nociceptive stimulation (at reflex threshold) of each of the contralateral fore- and hind paws in the control condition, during PAG/DRN stimulation, and ~10 min after a subcutaneous injection of morphine (1 mg/kg) followed by an additional dose of 2 mg/kg after ~30 min (*77*). Recordings in the S1 cortex were also made during 16 tactile stimulations of the same paws in the control condition and PAG/DRN stimulation. The cutaneous stimulation procedures were performed three times for each condition. Recordings of spontaneous activity were also measured during control, PAG/DRN stimulation, after the subcutaneous injection morphine during weeks 5 to 10. Recordings of spontaneous activity were performed during an inactive period, i.e., when the rats were not moving.

### Data analysis

All the analyses were performed automatically using in-house developed software in MATLAB or R (R Core Team, 2017) to avoid subjective biases.

#### Extracted evoked responses

Evoked cortical responses were extracted from the FP component and the multiunit firing rate estimate of neural recordings during nociceptive/tactile stimulation under different treatment conditions. Evoked FP responses to individual stimulus events were extracted (−0.2- to 0.8-s relative stimulus onset) from the low pass–filtered (<200 Hz) recording, sampled at 1 kHz, and preprocessed (50-Hz notch filter to suppress power line interference and stimulation artifacts, 10-ms second-degree Savitzky-Golay smoothing filter to suppress further stimulation artifacts, and linear trend removal to account for slow drift, excluding high-amplitude artifacts and overlapping events). The response was then estimated from the preprocessed event responses as the ensemble average across all events. The prestimulus average was subtracted, and the resulting response estimate was lastly smoothened with a 10-ms second-degree Savitzky-Golay filter.

Evoked neuronal responses were extracted from high pass–filtered (>300 Hz) wideband recordings, sampled at 40 kHz. Spike detection was performed (per channel) by applying a threshold of minus three times the estimated noise level. The noise level was estimated as the SD of the signal in a 5-s-long moving window, approximated by the scaled median absolute deviation of the recording (*78*). The moving estimator was applied to account for nonstationarities in noise level. Spikes with amplitude larger than 1 mV were considered to be artifacts. A peristimulus time histogram (PSTH) was calculated in 5-ms-long bins (−0.2- to 0.8-s relative stimulus onset), excluding erroneous overlapping events. The response estimate was obtained as the smoothed (20-ms Gaussian filter) *z* score normalized PSTH. The *z* score normalization was performed with respect to the prestimulus interval (−0.2 to 0 s) by subtracting the mean and dividing by the SD.

Intervals of interest (IOIs) for analyzing evoked FP and neuronal responses were determined automatically (and separately) for each combination of stimulus mode (nociceptive/tactile) and paw (fore-/hind paw) using the estimated neuronal responses in the control condition. The grand mean control neuronal response per animal was estimated as the average of all channel-averaged control neuronal responses across all experiments, excluding the recordings during hyperalgesia. The grand mean control neuronal responses from all animals were pooled, and a right-tailed sign test was applied in each 5-ms time bin (−0.2 to 0.8 s) to test whether the median neuronal response (across animals) *z* score was larger than zero. The response onset was taken as the first time point when the *P* value of the sign test indicated significance (*P* < 0.05) for at least four consecutive bins (20 ms), rounded down to the nearest multiple of 10 ms. Durations of IOIs were set as 500 and 60 ms from the detected onset time for nociceptive and tactile stimuli, respectively. The same IOIs were used for the analysis of the evoked FP. The resulting IOIs were consistent with previously reported IOIs (*45*, *48*, *79*).

#### Channel selection and response quantification

For each experiment, an automatic channel selection was performed to identify channels in the S1 array that exhibited the strongest control responses and thereby assumed to be good candidates for further analysis. This selection was performed separately for the cutaneous stimulus modes, paw, response type, and stimulus region (hyperalgesia experiments). The validity of this procedure was verified by also analyzing data from other channels.

FP responses were quantified in terms of the amplitude of the inverted response (i.e., negative amplitude). The time point (within the IOI) where the channel average reached its maximum negative amplitude was identified, and the channel with the largest negative amplitude in this time point was selected. Neuronal responses were quantified in terms of the average *z* score within the IOI. The average response estimate within the IOI was calculated for each channel for neuronal responses, and the channel with the highest average was selected.

#### Spontaneous neuronal activity

Spontaneous neuronal activity was quantified on all channels and based on spike detection in the same way as evoked responses but with the addition of stimulation artifact masking in the wideband signal before high-pass filtering. To avoid unintentional compensation for possible treatment-induced lowering of spontaneous activity and thereby presumably also lowering noise level and increased sensitivity to spikes, the control threshold of minus three times the estimated noise level was applied to all recordings on a given channel in each animal. The average firing rate was calculated as the total number of detected spikes on all channels divided by the total duration of the recording.

#### Impedance

Impedance measurements at 1 kHz from both S1 and PAG/DRN stimulation arrays were plotted over time to provide a qualitative measure of the stability of implants over time in terms of their electrical interface to the tissue (fig. S7). For PAG/DRN stimulation arrays, only microelectrodes used for stimulation were included. The impedance of a microelectrode was taken as the average of all measurements in each period. The measurements from individual microelectrodes were treated as independent measurements.

#### Withdrawal reflex rate

The number of withdrawals, defined as lifting the paw from the floor in response to nociceptive stimuli, was counted for each experimental session. The withdrawal rate was defined as the number of observed withdrawals divided by the total number of laser stimulations. For each animal, experiment, and condition, the withdrawal rate was taken as the average rate across all test repetitions.

#### Paw print intensity and walking speed

The speed and average of the mean paw print intensity obtained from five CatWalk runs were calculated for each rat. The ratio between the right and left hind paw mean intensities were calculated and used to measure paw pressure symmetry during gait.

### Statistical analysis

For all tests, a *P* < 0.05 was considered to indicate statistical significance. A detailed summary of the statistical analysis is provided in table S1.

#### Evoked responses and spontaneous neuronal activity

Evoked responses were plotted as the median and interquartile range across animals, and bin averages (10-ms bins) were compared over time. A Wilcoxon matched-pairs signed-rank test was conducted when comparing two treatments (e.g., “control” and “PAG/DRN stimulation”). When comparing three or more treatments [e.g., control, PAG/DRN stimulation, and “morphine 1 mg/kg”], a Friedman test was performed. If the Friedman test was significant, then a Dunn-Sidák multiple comparisons post hoc test was performed, and the significance level for each group versus the control was plotted. Evoked FP amplitude, averaged *z* score, and withdrawal reflex rate was compared between treatments using an automatically selected statistical test based on the number of treatments in the same way as when comparing response estimates over time, as above. Linear regression was used to analyze changes in the antinociceptive effect of PAG/DRN stimulation during a 1-hour continuous stimulation, using mean response amplitudes (evoked FP and average *z* score) and reflex rates obtained at four time points after initiation of PAG /DRN stimulation in each animal. Spontaneous neuronal activity was compared between all conditions using a Friedman test and a Dunn-Sidák multiple comparisons post hoc test.

#### Hyperalgesia assessment

Post- and pre-UVB measurements of skin blood flow were compared using a Wilcoxon matched-pairs signed-rank test.

#### Paw print and gait analysis

Comparing the groups (pre-UVB versus pre–UVB-PAG/DRN stimulation and post-UVB versus post–UVB-PAG/DRN stimulation) of paw print intensity ratio and gait speed was performed with a Wilcoxon matched-pairs signed-rank test. To assess whether or not each individual condition was associated with symmetry in right and left paw print intensity, a *t* test was performed on the intensity ratios for each condition with the hypothesis that the mean equals one. Before performing the *t* test, each set of paw print intensities was tested for normality using the Lilliefors’ goodness-of-fit test of composite normality.

### Excluded rats

One rat with stimulation probe placement outside the PAG/DRN region and one rat with disruption of the contact during surgery were excluded. During the 1-hour-long PAG/DRN stimulation weeks 4 to 5 and spontaneous activity recordings, one rat had to be excluded because of external sound disturbances. During CatWalk experiments, one rat lost the implant and thus had to be terminated during week 7. One rat had to be excluded from the last test during weeks 10 to 11 because of grounding failure in the S1 recording array.

## Supplementary Materials

### This PDF file includes:

Cresyl violet staining for stimulation electrode localization Figs. S1 to S9 Table S1 Legend for movie S1

## Notes

View/request a protocol for this paper from *Bio-protocol*.
