# Supplementary material for: 3D microelectrode cluster and stimulation paradigm yield powerful analgesia without noticeable adverse effects
Source: Sci Adv. 2021 Oct 8;7(41):eabj2847. doi: 10.1126/sciadv.abj2847 (PMC8500508; doi:10.1126/sciadv.abj2847)
Supplement: Supplementary file 1 — Cresyl violet staining for stimulation electrode localization Figs. S1 to S9 Table S1 Legend for movie S1 [file sciadv.abj2847_sm.pdf]

Science Advances
AAAS

Supplementary Materials for

3D microelectrode cluster and stimulation paradigm yield powerful analgesia without noticeable adverse effects

Matilde Forni*, Palmi Thor Thorbergsson, Jonas Thelin, Jens Schouenborg*

*Corresponding author. Email: matilde.forni@med.lu.se (M.F.); jens.schouenborg@med.lu.se (J.S.)

Published 8 October 2021, *Sci. Adv.* **7**, eabj2847 (2021)
DOI: 10.1126/sciadv.abj2847

The PDF file includes:

Cresyl violet staining for stimulation electrode localization
Figs. S1 to S9
Table S1
Legend for movie S1

Other Supplementary Material for this manuscript includes the following:

Movie S1

Cresyl violet staining for stimulation electrode localization

The implant detached in one rat, prompting an immediate termination. The rat was transcardially perfused under deep anesthesia (overdose of pentobarbital) with ~100 ml of room temperature 0.9% saline solution followed by ~300 ml of ice-cold 4% paraformaldehyde in 0.1 M phosphate buffer at pH 7.4. After the skull removal, the brain was put in 20% sucrose solution for cryoprotection until the brain sunk in the solution, snap-frozen in 2-methylbutane solution at -78 °C, stored at -80 °C, cryosectioned (30 mm slices, Microm HM 560, Microm GmbH, Walldorf, Germany) and put onto Super Frost® plus slides (Menzel-Gläser, Thermo Scientific). The slices were immersed in a 1:1 solution of ethanol/chloroform overnight, then in 99.5% ethanol for two minutes, 95% ethanol solutions for two minutes, and finally in MilliQ water for two minutes. The sections were then stained with cresyl violet (Sigma). Re-immersed in MilliQ water for five minutes, 95% ethanol for six minutes, 99.5% ethanol for five minutes, and with xylene (two times for 5 minutes) and coverslipped with DPX mounting media (Fluka, Germany). Finally, the probe location in the PAG/DRN area was verified using a 4x microscope (Olympus SZX2-TR30, Olympus corporation).

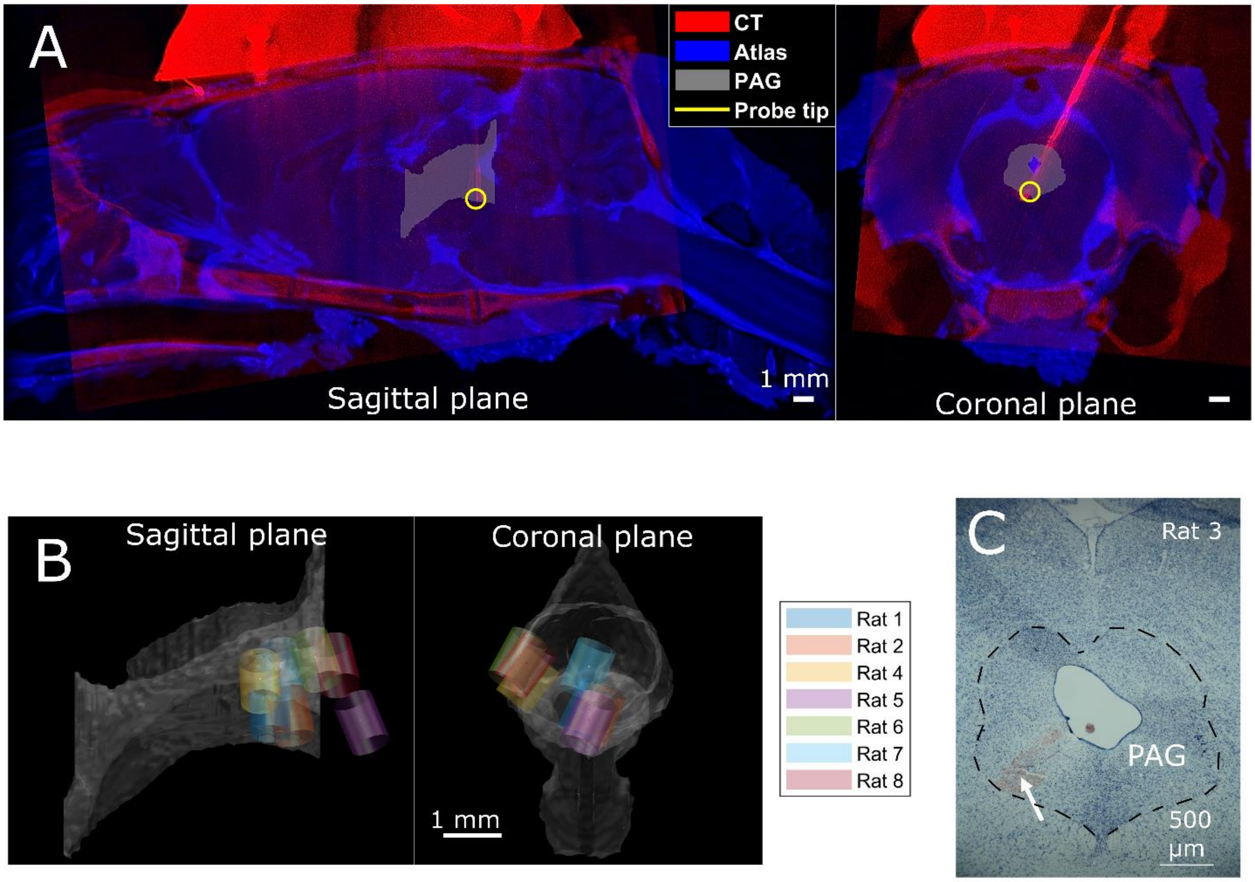

Fig. S1. Computed tomography to locate the placement of stimulation probe *in vivo*. (**A**) An example of a CT 3D reconstructed image superimposed on a standardized Waxholm rat brain atlas shows the stimulation probe tip within the PAG area. (**B**) Pooled placements of the electrode contact areas (the distal part of the probe of each of the 7 CT scanned animals is represented by a cylinder corresponding to the average spread of microelectrode contacts, see Methods) with respect to the contour of the superimposed standardized Waxholm atlas of PAG (same greyish area as in A). (**C**) Histological coronal section stained with cresyl violet and showing a slight hemorrhage remnant (brownish stain indicated by arrow) of the distal part of the extracted probe (further sections showed more proximal parts of the probe).

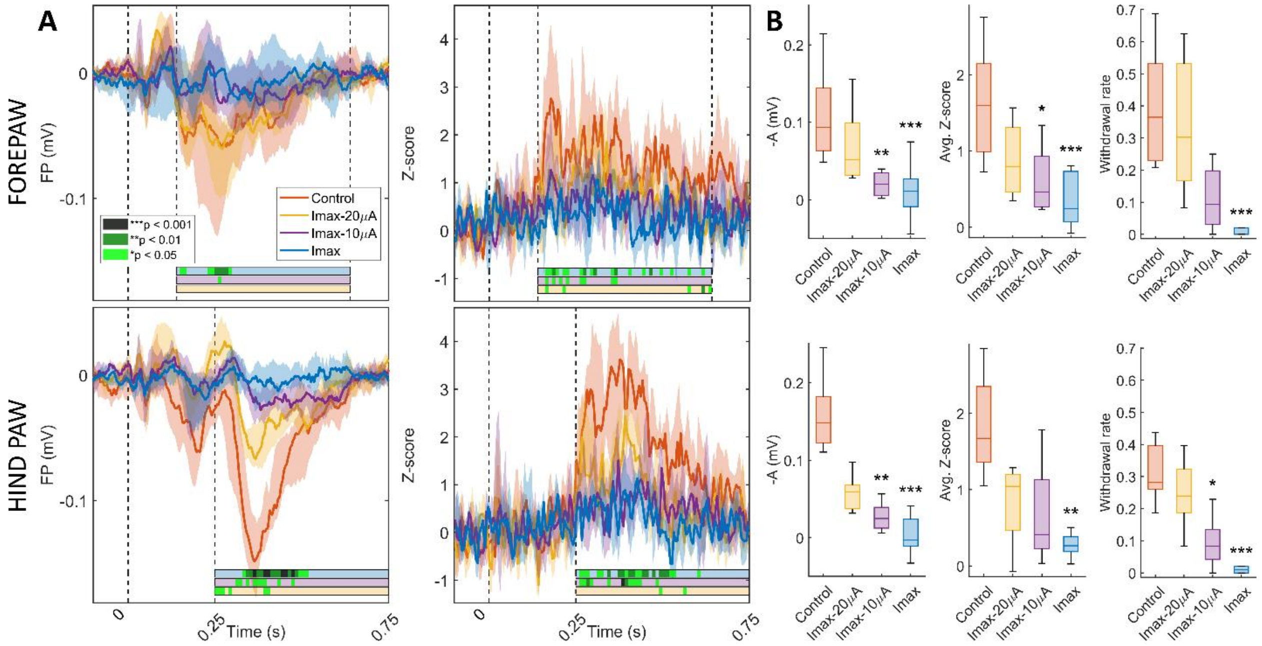

Fig. S2. Current-dependent effects of PAG/DRN stimulation on nociceptive-evoked cortical FP/neural responses and nociceptive withdrawal reflexes. Three different stimulation currents (Imax, Imax-10 µA, and Imax-20 µA) were used. (A) The colored lines and corresponding shaded areas indicate the median and interquartile range, respectively, of nociceptive-evoked FP amplitude (left) and the neuronal responses (Z-score, right) recorded in the contralateral fore and hind paw S1 cortex in control and during PAG/DRN stimulation (n=8). The horizontal color-coded bars under each graph represent statistical significance level (Friedman's test with Dunn-Sidák post-hoc) when comparing amplitudes during PAG/DRN stimulation with control in the interval of interest (IOI, forepaw 140-640 ms post-stimulus, hind paw 250-750 ms post-stimulus, IOI indicated by vertical dashed lines). For each 10 ms bin time, p-values are color-labeled, as explained in the upper left box. (B) The box and whisker plots (median and quartiles) represent the inverted nociceptive-evoked FP response amplitude at the time point of maximum control response (left); the average Z-score within the IOI (middle) and the withdrawal rate (right) in fore and hind paw (n=8; *** =p< 0.001, ** =p< 0.01, Friedman's test with Dunn-Sidák post-hoc test). FP, field potential voltage; -A, inverted response amplitude; Avg. Z-score averaged Z-score in the IOI.

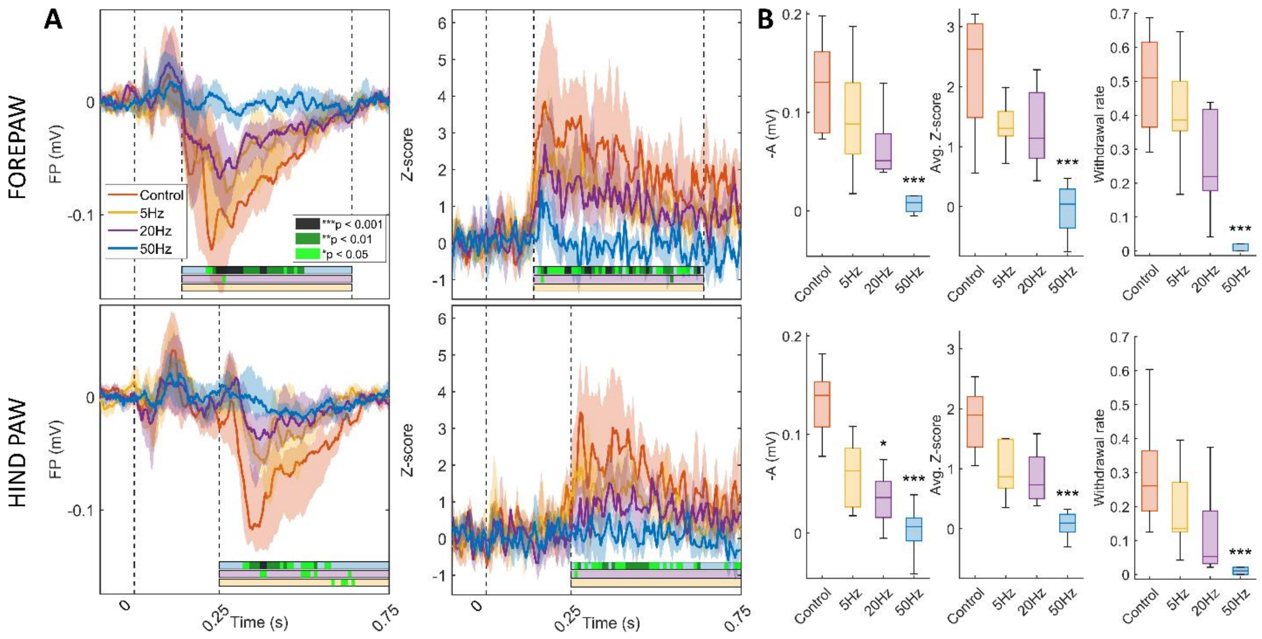

Fig. S3. Frequency-dependent effects of PAG/DRN stimulation on nociceptive-evoked cortical FP/neuronal responses and nociceptive withdrawal reflexes. Three different stimulation frequencies were used; 5, 20, and 50Hz. (A) The colored lines indicate the median and interquartile range of nociceptive-evoked FPs amplitude (left) and neuronal responses (Z-score, right) in the fore and hind paws S1 cortex across the animals (n=8). Graphics and statistics as in fig S1. (B) Box and whisker plots (median and quartiles) represent the inverted nociceptive-evoked FP response amplitude at the time point of maximum control response (left), the average neuronal Z-score within the IOI (middle), and the withdrawal rate (right) in fore and hind paw (n=8; *** =p< 0.001, ** =p< 0.01, Friedman's test with Dunn-Sidák post-hoc test). FP, field potential voltage; -A, inverted response amplitude; Avg. Z-score averaged Z-score in the IOI.

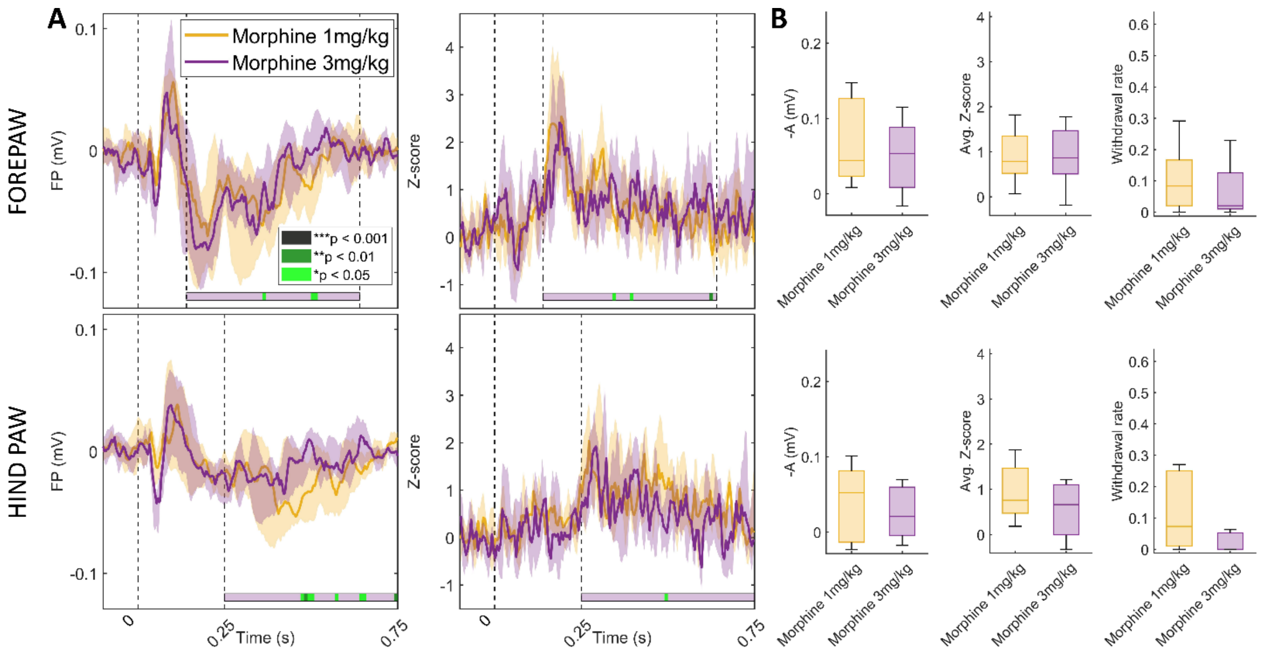

Fig. S4. Minimal effects of adding 2 mg/kg, s.c. morphine to the initial dose of 1 mg/kg s.c. on nociceptive-evoked cortical FP/neuronal responses and nociceptive withdrawal reflexes. (**A**) The colored lines indicate the median and interquartile range of nociceptive-evoked FPs amplitude (left) and neuronal responses (Z-score, right) in the fore and hind paws S1 cortex across the animals (n=8). The shaded area around the median values represents the interquartile range. The recordings are shown from -0.1 to 0.750 s with respect to stimulus onset. The horizontal color-coded bars under each graph represent the statistical significance level (Wilcoxon matched-pairs signed-rank) of the difference between amplitudes during PAG/DRN stimulation and control (bin size=10 ms; interval of interest (IOI) =0.140-0.640 s post-stimulus for the forepaw and 0.250-0.750 s post-stimulus for the hind paw). (**B**) Box and whisker plots (median and quartiles) represent the inverted nociceptive-evoked FP response amplitude at the time point of maximum control response (left), the average neuronal Z-score within the IOI (middle), and the withdrawal rate (right) in fore and hind paw (n=8; Wilcoxon matched-pairs signed-rank). FP, field potential voltage; -A, inverted response amplitude; Avg. Z-score averaged Z-score in the IOI.

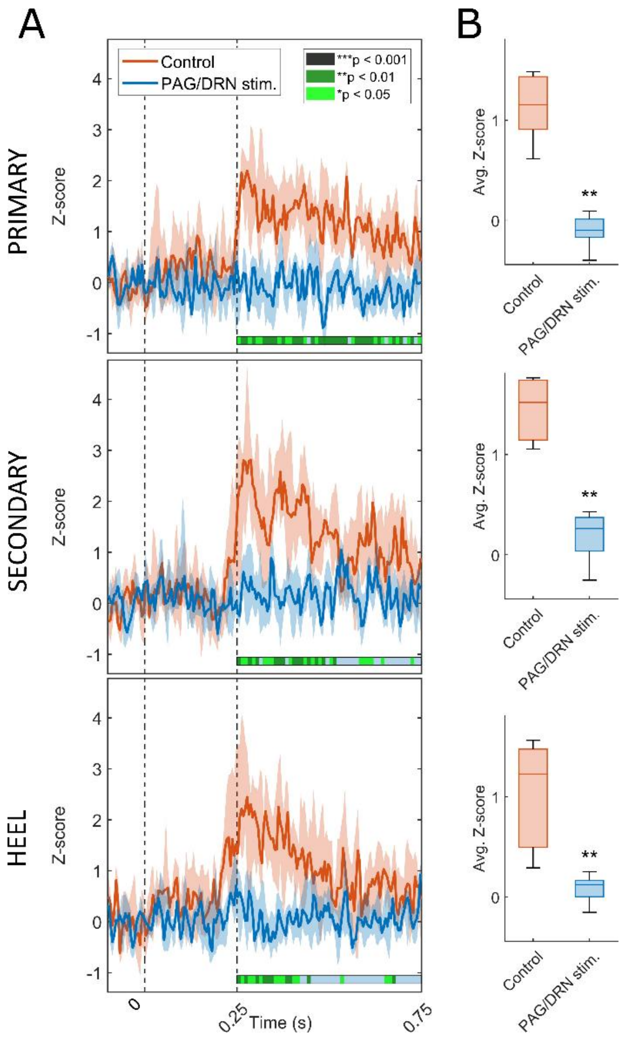

Fig. S5. Abolished neuronal responses in primary somatosensory cortex during PAG/DRN stimulation during hyperalgesia. (**A**) The colored lines indicate the median and interquartile range of the neuronal responses (Z-score) in the fore and hind paws S1 cortex across the animals (n=8). The shaded area around the median values represents the interquartile range. The recordings are shown from -0.1 to 0.750 s with respect to stimulus onset. The horizontal color-coded bars under each graph represent the statistical significance level (Wilcoxon matched-pairs signed-rank) of the difference between Z-scores during PAG/DRN stimulation and control (bin size=10 ms; interval of interest (IOI) =0.140-0.640 s post-stimulus for the forepaw and 0.250-0.750 s post-stimulus for the hind paw). (**B**) Box and whisker plots (median and quartiles) represent the averaged neuronal Z-score within the IOI in fore and hind paw (n=8; Wilcoxon matched-pairs signed-rank). Avg. Z-score averaged Z-score in the IOI.

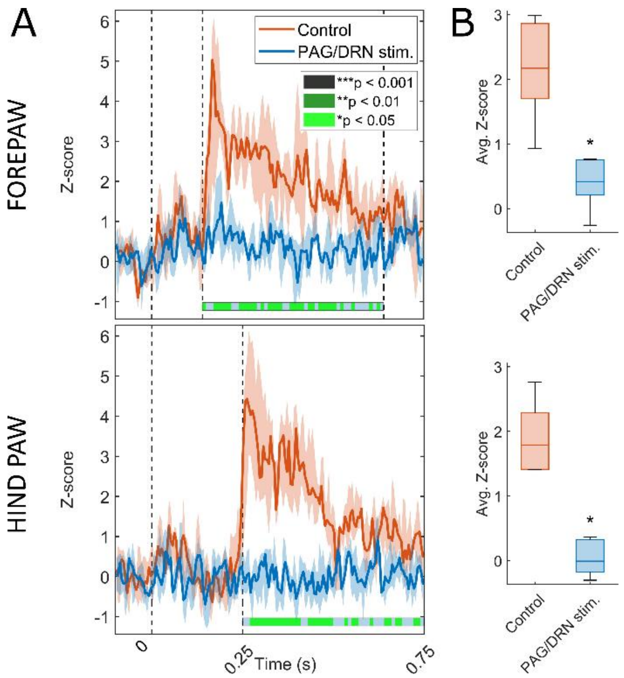

Fig. S6. Analgesic effects on neuronal responses induced by PAG/DRN stimulation are still powerful in the long-term follow-up. Graphics and statistics concerning neuronal responses as in fig. S4 (n=6).

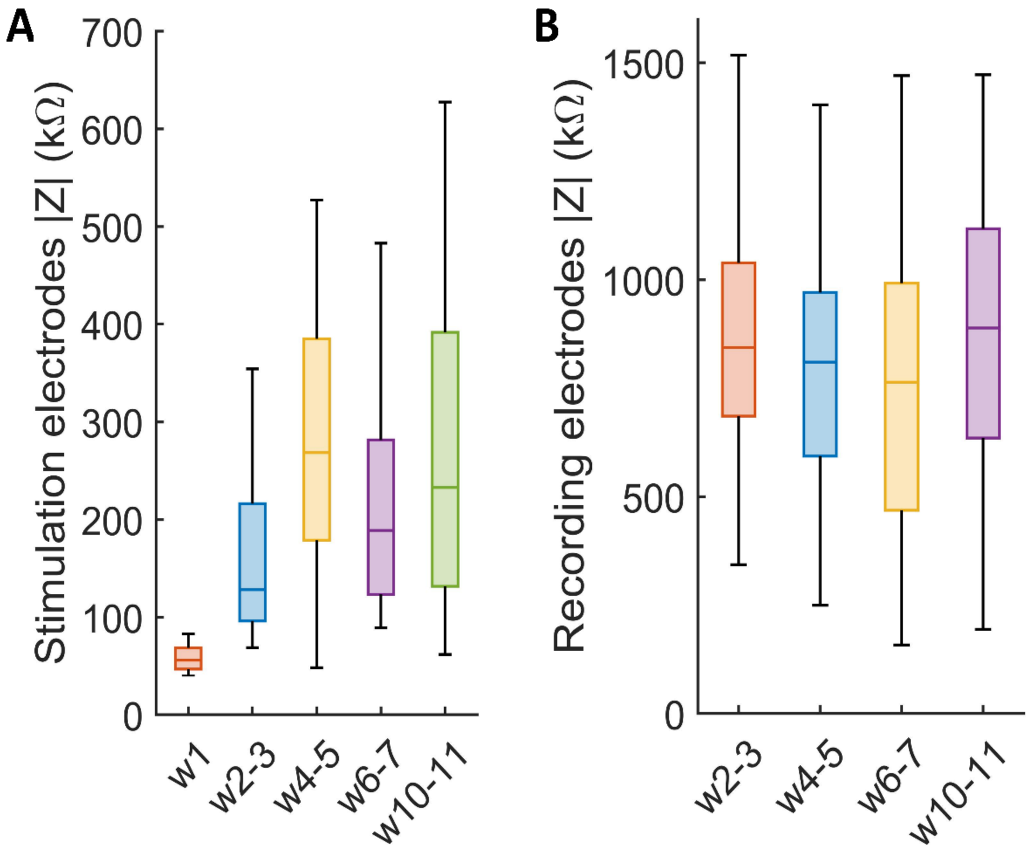

Fig. S7. Electrode impedance throughout the study. (**A**) Impedance of PAG/DRN stimulation microelectrodes and (**B**) S1 cortical recording microelectrodes measured at different time intervals post-implantation (**A**: n=17, 33, 33, 29, 30; **B**: n=48, 48, 48, 42 for the respective time intervals). All impedance measurements were made at 1 kHz. The impedance of the stimulation microelectrodes was performed before PAG/DRN stimulation. w, week; |Z|, impedance magnitude.

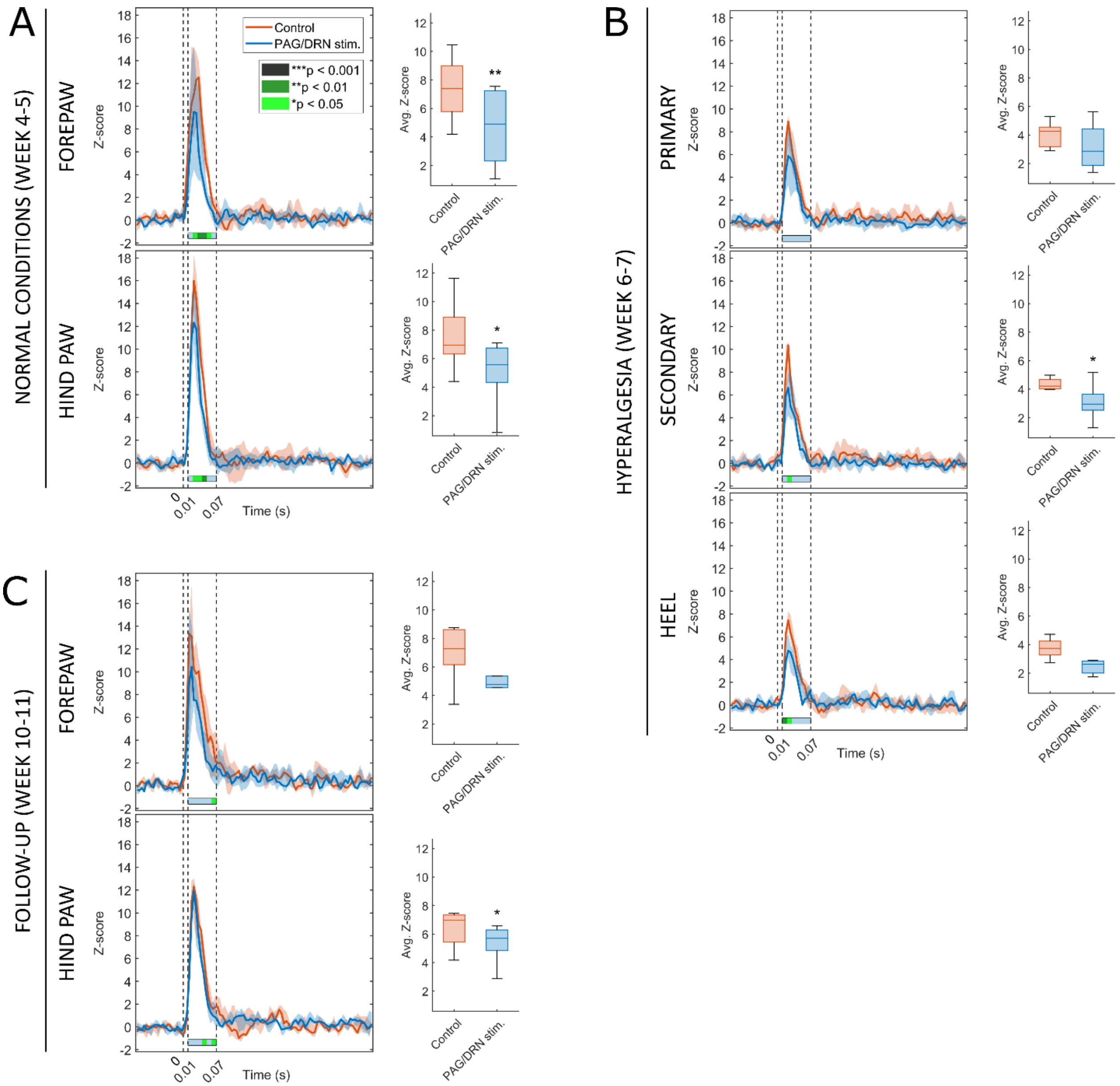

Fig. S8. Minor effects of PAG/DRN stimulation on tactile-evoked cortical neuronal responses in control conditions and during hyperalgesia. The median and interquartile range of neuronal responses in the left panels of figures (**A**), (**B**), and (**C**) throughout the study during the normal condition in fore and hind paw weeks 4-5 (**A**; n=8) and weeks 10-11 (**C**; n=6) and during hyperalgesia weeks 6-7 (**B**; n=8, hind paw). Time-axis from -0.1 s to 0.4 s stimulus onset. The horizontal bars under each graph provide the statistical significance of the difference between PAG/DRN stimulation and the control (bin size=10 ms; IOI = 0.01-0.07 s post-stimulus represented by the second and the third vertical dashed lines; Wilcoxon matched-pairs signed-rank). Box and whisker plots (median and quartiles) in the right panel of the figure (**A**), (**B**), and (**C**) representing the average Z-score within the IOI average Z-score within the IOI (right) in fore and hind paw or primary, secondary or heel area during hyperalgesia (*=p< 0.05; Wilcoxon matched-pairs signed-rank). Avg. Z-score averaged Z-score in the time IOI.

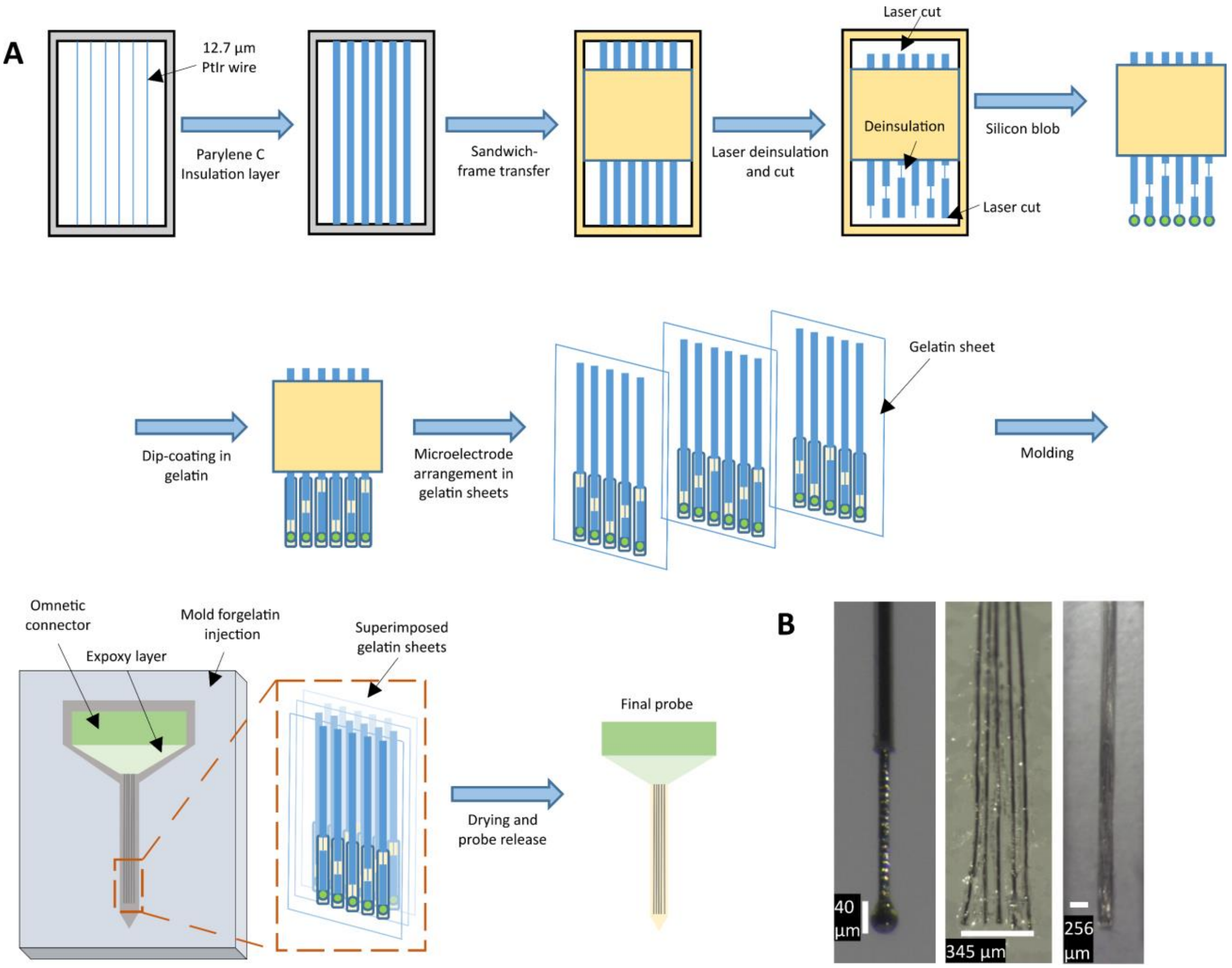

Fig. S9. Manufacturing steps to produce a stimulation microelectrode probe. (**A**) Schematic of the manufacturing of the PAG/DRN stimulation probe. (**B**) Representative images of parylene C insulated wire, with deinsulation of 300 µm from the tip and a distal silicon blob (left); the arrangement of wires and freeze-fixated flake of gelatin (middle); final probe (right). Scale bars are indicated in each image. Photo Credit: Matilde Forni, Lund University, Sweden.

Table S1.

Detailed statistical analysis descriptions. -A, inverted response amplitude; Avg. Z-score, averaged Z-score in the interval of interest; W, W-value; Df, degree of freedom; F, F-value; RSq, R-Square; ChiSq, Chi-Square; t-stat, t-statistic; k-stat, k-statistic; p, p-value.

Figure 3B

Forepaw	n (animals)	Test type	W	p
-A	8	Wilcoxon matched-pairs signed-rank	36	0.0078
Avg. Z-score	8	Wilcoxon matched-pairs signed-rank	36	0.0078
Withdrawal rate	8	Wilcoxon matched-pairs signed-rank	36	0.0078

Hind paw	n (animals)	Test type	W	p
-A	8	Wilcoxon matched-pairs signed-rank	36	0.0078
Avg. Z-score	8	Wilcoxon matched-pairs signed-rank	36	0.0078
Withdrawal rate	8	Wilcoxon matched-pairs signed-rank	36	0.0078

Figure 3C

Forepaw	n*	Test type	Df Regression	Df Residual	F	RSq	p
-A	7 x 4	Single linear regression	1	26	0.0140	0.0005	0.9066
Avg. Z-score	7 x 4	Single linear regression	1	26	6.3178	0.1954	0.0184
Withdrawal rate	7 x 4	Single linear regression	1	26	9.8156	0.2740	0.0042

Hind paw	n*	Test type	Df Regression	Df Residual	F	RSq	p
-A	7 x 4	Single linear regression	1	26	2.1545	0.0765	0.1541
Avg. Z-score	7 x 4	Single linear regression	1	26	0.3559	0.0135	0.5559
Withdrawal rate	7 x 4	Single linear regression	1	26	12.9327	0.3322	0.0013

* n = animals x timepoints

Figure 4B

Forepaw	n (animals)	Test type	Df	ChiSq	p
-A	8	Friedman with Dunn-Sidák post-hoc	2	10.75	0.0046
Avg. Z-score	8	Friedman with Dunn-Sidák post-hoc	2	6.25	0.0439
Withdrawal rate	8	Friedman with Dunn-Sidák post-hoc	2	10.51	0.0052

Hind paw	n (animals)	Test type	Df	ChiSq	p
-A	8	Friedman with Dunn-Sidák post-hoc	2	7.75	0.0208
Avg. Z-score	8	Friedman with Dunn-Sidák post-hoc	2	16	0.0003
Withdrawal rate	8	Friedman with Dunn-Sidák post-hoc	2	12.19	0.0022

Figure 4C

	n (animals)	Test type	Df	ChiSq	p
Spontaneous activity	7	Friedman with Dunn-Sidák post-hoc	2	12.28	0.0021

Figure 5B

	n (animals)	Test type	W	p
Blood flow	8	Wilcoxon matched-pairs signed-rank	36	0.0078

Figure 5D

Primary	n (animals)	Test type	W	p
-A	8	Wilcoxon matched-pairs signed-rank	36	0.0078
Withdrawal rate	8	Wilcoxon matched-pairs signed-rank	36	0.0078

Secondary	n (animals)	Test type	W	p
-A	8	Wilcoxon matched-pairs signed-rank	36	0.0078
Withdrawal rate	8	Wilcoxon matched-pairs signed-rank	36	0.0078

Heel	n (animals)	Test type	W	p
-A	8	Wilcoxon matched-pairs signed-rank	36	0.0078
Withdrawal rate	8	Wilcoxon matched-pairs signed-rank	36	0.0078

Figure 6B

Pre-UVB	n (animals)	Test type	W	p
Averaged speed	7	Wilcoxon matched-pairs signed-rank	4	0.8125

Post-UVB	n (animals)	Test type	W	p
Averaged speed	7	Wilcoxon matched-pairs signed-rank	16	0.2188

Figure 6C

Control Vs. PAG-DRN stim

Pre-UVB	n (animals)	Test type	W	p
Mean Intensity	7	Wilcoxon matched-pairs signed-rank	2	1

Post-UVB	n (animals)	Test type	W	p

Mean Intensity	7	Wilcoxon matched-pairs signed-rank	2	1

Normality

Pre-UVB	n (animals)	Test type	K-stat	p
Control	7	Lilliefors composite goodness-of-fit	0.2292	0.3235
PAG-DRN stim	7	Lilliefors composite goodness-of-fit	0.2333	0.3000

Post-UVB	n (animals)	Test type	K-stat	p
Control	7	Lilliefors composite goodness-of-fit	0.2280	0.3305
PAG-DRN stim	7	Lilliefors composite goodness-of-fit	0.2935	0.0688

Symmetry

Pre-UVB	n (animals)	Test type	T-stat	Df	p
Control	7	One-sample t-test	-0.4544	6	0.6655
PAG-DRN stim	7	One-sample t-test	-0.3957	6	0.7060

Post-UVB	n (animals)	Test type	T-stat	Df	p
Control	7	One-sample t-test	-7.8059	6	2.3312e-04
PAG-DRN stim	7	One-sample t-test	-2.7057	6	0.0353

Figure 6D

Control Vs. PAG-DRN stim

Pre-UVB	n (animals)	Test type	W	p
Mean Intensity	7	Wilcoxon matched-pairs signed-rank	11	0.6875

Post-UVB	n (animals)	Test type	W	p
Mean Intensity	7	Wilcoxon matched-pairs signed-rank	11	0.6875

Normality

Pre-UVB	n (animals)	Test type	K-stat	p
Control	7	Lilliefors composite goodness-of-fit	0.1626	0.5000
PAG-DRN stim	7	Lilliefors composite goodness-of-fit	0.2377	0.2761

Post-UVB	n (animals)	Test type	K-stat	p
Control	7	Lilliefors composite goodness-of-fit	0.1645	0.5000
PAG-DRN stim	7	Lilliefors composite goodness-of-fit	0.2117	0.4443

Symmetry

Pre-UVB	n (animals)	Test type	T-stat	Df	p
Control	7	One-sample t-test	0.1521	6	0.8841
PAG-DRN stim	7	One-sample t-test	0.7055	6	0.5070

Post-UVB	n (animals)	Test type	T-stat	Df	p
Control	7	One-sample t-test	-1.5717	6	0.1671
PAG-DRN stim	7	One-sample t-test	-1.2441	6	0.2598

Figure 7B

Forepaw	n (animals)	Test type	W	p
-A	6	Wilcoxon matched-pairs signed-rank	21	0.0313
Withdrawal rate	6	Wilcoxon matched-pairs signed-rank	21	0.0313

Hind paw	n (animals)	Test type	W	p
-A	6	Wilcoxon matched-pairs signed-rank	21	0.0313
Withdrawal rate	6	Wilcoxon matched-pairs signed-rank	21	0.0313

Figure 8A

Forepaw	n (animals)	Test type	W	p
-A	8	Wilcoxon matched-pairs signed-rank	34	0.0234

Hind paw	n (animals)	Test type	W	p
-A	8	Wilcoxon matched-pairs signed-rank	32	0.0547

Figure 8B

Primary	n (animals)	Test type	W	p
-A	8	Wilcoxon matched-pairs signed-rank	24	0.4609

Secondary	n (animals)	Test type	W	p
-A	8	Wilcoxon matched-pairs signed-rank	30	0.1094

Heel	n (animals)	Test type	W	p
-A	8	Wilcoxon matched-pairs signed-rank	33	0.0391

Figure 8C

Forepaw	n (animals)	Test type	W	p
-A	6	Wilcoxon matched-pairs signed-rank	18	0.1563

Hind paw	n (animals)	Test type	W	p
-A	6	Wilcoxon matched-pairs signed-rank	20	0.0625

Figure S2B

Forepaw	n (animals)	Test type	Df	ChiSq	p
-A	8	Friedman with Dunn-Sidák post-hoc	3	22.2	5.93e-05
Avg. Z-score	8	Friedman with Dunn-Sidák post-hoc	3	15.9	0.0012
Withdrawal rate	8	Friedman with Dunn-Sidák post-hoc	3	19.07	0.0003

Hind paw	n (animals)	Test type	Df	ChiSq	p
-A	8	Friedman with Dunn-Sidák post-hoc	3	17.55	0.0005

Avg. Z-score	8	Friedman with Dunn-Sidák post-hoc	3	12.6	0.0056
Withdrawal rate	8	Friedman with Dunn-Sidák post-hoc	3	21.08	0.0001

Figure S3B

Forepaw	n (animals)	Test type	Df	ChiSq	p
-A	8	Friedman with Dunn-Sidák post-hoc	3	20.85	0.0001
Avg. Z-score	8	Friedman with Dunn-Sidák post-hoc	3	18.15	0.0004
Withdrawal rate	8	Friedman with Dunn-Sidák post-hoc	3	20.85	0.0001

Hind paw	n (animals)	Test type	Df	ChiSq	p
-A	8	Friedman with Dunn-Sidák post-hoc	3	17.55	0.0005
Avg. Z-score	8	Friedman with Dunn-Sidák post-hoc	3	16.2	0.0010
Withdrawal rate	8	Friedman with Dunn-Sidák post-hoc	3	18.54	0.0003

Figure S4B

Forepaw	n (animals)	Test type	W	p
-A	8	Wilcoxon matched-pairs signed-rank	23	0.5469
Avg. Z-score	8	Wilcoxon matched-pairs signed-rank	22	0.6406
Withdrawal rate	8	Wilcoxon matched-pairs signed-rank	15.5	0.3438

Hind paw	n (animals)	Test type	W	p
-A	8	Wilcoxon matched-pairs signed-rank	22	0.6406
Avg. Z-score	8	Wilcoxon matched-pairs signed-rank	23	0.5469
Withdrawal rate	8	Wilcoxon matched-pairs signed-rank	19.5	0.0938

Figure S5B

Primary	n (animals)	Test type	W	p
Avg. Z-score	8	Wilcoxon matched-pairs signed-rank	36	0.0078

Secondary	n (animals)	Test type	W	p
Avg. Z-score	8	Wilcoxon matched-pairs signed-rank	36	0.0078

Primary	n (animals)	Test type	W	p
Avg. Z-score	8	Wilcoxon matched-pairs signed-rank	36	0.0078

Figure S6B

Forepaw	n (animals)	Test type	W	p
Avg. Z-score	6	Wilcoxon matched-pairs signed-rank	21	0.0313

Hind paw	n (animals)	Test type	W	p
Avg. Z-score	6	Wilcoxon matched-pairs signed-rank	21	0.0313

Figure S8A

Forepaw	n (animals)	Test type	W	p
Avg. Z-score	8	Wilcoxon matched-pairs signed-rank	36	0.0078

Hind paw	n (animals)	Test type	W	p
Avg. Z-score	8	Wilcoxon matched-pairs signed-rank	35	0.0156

Figure S8B

Primary	n (animals)	Test type	W	p
Avg. Z-score	8	Wilcoxon matched-pairs signed-rank	27	0.25

Secondary	n (animals)	Test type	W	p
Avg. Z-score	8	Wilcoxon matched-pairs signed-rank	35	0.0156

Heel	n (animals)	Test type	W	p
Avg. Z-score	8	Wilcoxon matched-pairs signed-rank	31	0.0781

Figure S8C

Forepaw	n (animals)	Test type	W	p
Avg. Z-score	6	Wilcoxon matched-pairs signed-rank	20	0.0625

Hind paw	n (animals)	Test type	W	p
Avg. Z-score	6	Wilcoxon matched-pairs signed-rank	21	0.0313

Movie S1.
3D reconstruction of PAG and probe location for each rat.
